# Synthesis of Novel 2-(Isopropylamino)thiazol-4(5*H*)-one Derivatives and Their Inhibitory Activity of 11β-HSD1 and 11β-HSD2 in Aspect of Carcinogenesis Prevention

**DOI:** 10.3390/molecules25184233

**Published:** 2020-09-15

**Authors:** Daria Kupczyk, Renata Studzińska, Rafał Bilski, Szymon Baumgart, Renata Kołodziejska, Alina Woźniak

**Affiliations:** 1 Department of Medical Biology and Biochemistry, Faculty of Medicine, Collegium Medicum in Bydgoszcz, Nicolaus Copernicus University in Toruń, 85-092 Bydgoszcz, Poland; rafal.bilski@cm.umk.pl (R.B.); renatak@cm.umk.pl (R.K.); alina-wozniak@wp.pl (A.W.); 2 Department of Organic Chemistry, Faculty of Pharmacy, Collegium Medicum in Bydgoszcz, Nicolaus Copernicus University in Toruń, 85-089 Bydgoszcz, Poland; szymonbaumgart10@gmail.com

**Keywords:** carcinogenesis, anti-cancer therapy, glucocorticoids, thiazolone derivatives, enzyme inhibition, 11β-hydroxysteroid dehydrogenase, cell proliferation

## Abstract

Glucocorticoid metabolism at the tissue level is regulated by two isoenzymes 11β-hydroxysteroid dehydrogenase (11β-HSD), which mutually convert biologically active cortisol and inactive cortisone. Recent research is focused on the role of 11β-HSD1 and 11β-HSD2 as autocrine factors of tumor cell proliferation and differentiation. Herein, we report the synthesis of novel 2-(isopropylamino)thiazol-4(5*H*)-one derivatives and their inhibitory activity for 11β-HSD1 and 11β-HSD2. The derivative containing the spiro system of thiazole and cyclohexane rings shows the highest degree of 11β-HSD1 inhibition (54.53% at 10 µM) and is the most selective inhibitor of this enzyme among the tested compounds. In turn, derivatives containing ethyl and *n*-propyl group at C-5 of thiazole ring inhibit the activity of 11β-HSD2 to a high degree (47.08 and 54.59% at 10 µM respectively) and are completely selective. Inhibition of the activity of these enzymes may have a significant impact on the process of formation and course of tumors. Therefore, these compounds can be considered as potential pharmaceuticals supporting anti-cancer therapy.

## 1. Introduction

Cancer is a major public health problem and a leading cause of death worldwide. Increased investment in basic and clinical research to find the metabolic processes underlying carcinogenesis would help to advance treatment options for patients [1]. In the course of numerous studies carried out over the years, researchers have managed to establish that the formation and development of neoplasms is influenced by psychophysiological factors such as stress, depression, or social isolation. The results indicate that the psychosocial factors associated with stress, predispose to a higher incidence of cancer in healthy people [2,3,4,5]. The main physiological pathway related to chronic stress response is the hypothalamic–pituitary–adrenal (HPA) axis. Both acute and chronic stress influence the glucocorticoid response, which leads to changes in diurnal secretion of cortisol. It is the chronic stress, the feeling of anxiety or the overwhelming hopelessness that seems to have a much greater impact on the phenomenon of cancer development than the cases of sudden and short-term events that may cause temporary stress. Therefore, it is indicated that the permanent stimulation of negative affect pathways may constitute the development of neoplasm [6,7,8]. In addition, there are many biochemical markers that can be used to assess the level of stress and exposure to its effects in humans. Among such compounds, we can distinguish hormones such as epinephrine or cortisol [9,10]. Cortisol, as a representative of glucocorticoids, is a hormone produced by the adrenal cortex that affects many metabolic factors in the body.

**Scheme 1:**
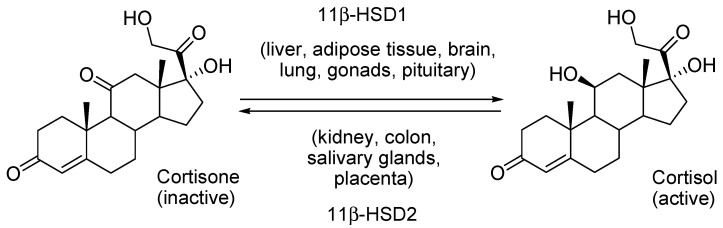
The physiological role of the two isoforms of 11β-HSD.

The most important factor affecting its secretion and access to target cells is a microsomal enzyme named 11β-hydroxysteroid dehydrogenase (11β-HSD). This enzyme exists in two isoforms 11β-HSD1 and 11β-HSD2. The first one catalyzes the reaction that converts biochemically inactive cortisone into its active counterpart cortisol. On the other hand, 11β-HSD2 is used catalytically in a reverse reaction, in a way of inactivation of cortisol, transforming it into cortisone (Scheme 1) [11,12]. Chronic stress causes an increase in the activity of 11β-HSD1, which leads to an increase in the level of cortisol in the body [13]. Elevated cortisol levels effectively reduce the effectiveness of the immune system [14,15]. On the other hand, in the process of neoplasm, effective recognition of pathological changes by the immune system is a key phenomenon in therapy and stopping the development of the disease. Particularly high activity of NK cells (Natural Killer) inhibits the formation and development of metastases in patients through the activation of NKG2D and DNAM-1 receptors [16]. The 11β-HSD1 activity appears to be directly related to the level of NK cells. Lowering the enzyme activity increases the number of NK cells, and thus provides a more effective immune response [17].

In turn, 11β-HSD2 is involved in carcinogenesis, tumor progression and metastasis along with its role in inflammation and hypertension regulation [18,19]. There are studies that have shown the promotion of tumor progression and metastasis by this enzyme, which may be due to its physiological function of inactivating glucocorticoids. Moreover, pharmacological inhibition of 11β-HSD2 prevented tumor formation, growth, and metastasis. The studies have shown that 11β-HSD2 induces mobility of colorectal cancer cells [20]. The AKT pathway activation plays a significant role in cell metabolism, growth, and proliferation [21]. However, the other mechanisms by which 11β-HSD2 metastasis are unknown. Therefore, we are looking for selective inhibitors of glucocorticoids that can play a therapeutic role in the process of carcinogenesis and provide the basis for understanding the mechanisms underlying the impact of stress and glucocorticoid action on cancer development. This could prove to be a novel approach in cancer chemoprevention.

**Scheme 2:**
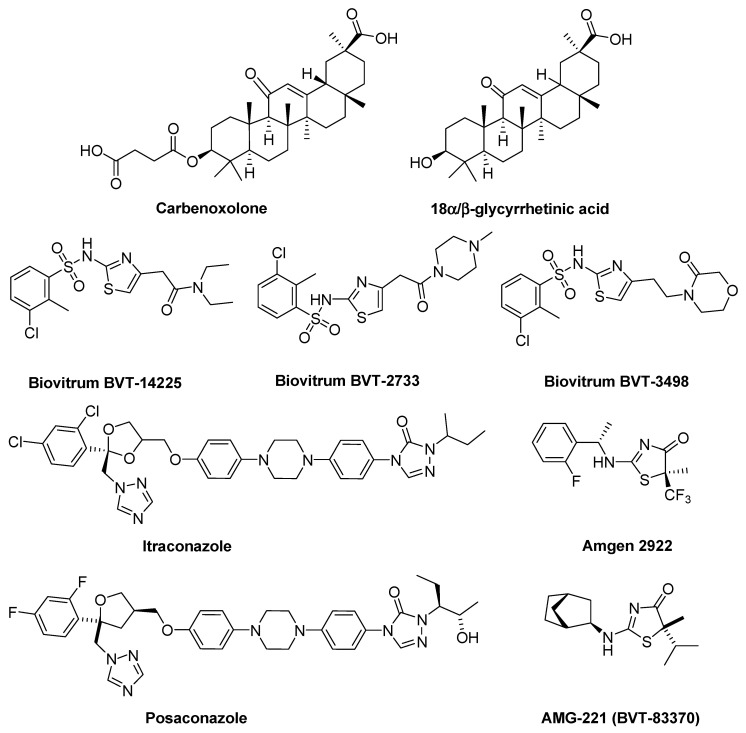
The inhibitors of 11β-HSD.

Carbenoxolone (CBX, 18β-glycyrrhetinic acid 3β-*O*-hemisuccinate) is the known inhibitor of 11β-HSD1. It is the hemisuccinate ester derivative of glycyrrhetinic acid, a natural product found in liquorice root. Not only does carbenoxolone inhibit 11β-HSD1, but it is also—To a lesser degree, though an inhibitor of 11β-hydroxysteroid dehydrogenase type 2. Likewise, the glycyrrhetinic acid (18β-glycyrrhetinic acid, 18β-GA 1) itself is potent non-selective inhibitor of both 11β-HSD1 and 11β-HSD2, although it inhibits the type 2 enzyme to a higher degree at different concentrations. [22,23]. Non-selective activity of both carbenoxolone and glycyrrhetinic acid affects their limited range of clinical applications, prompting a search for new compounds—selective inhibitors of 11β-HSD (Scheme 2).

The group of non-steroidal 11β-HSD1 inhibitors includes, inter alia, 4-substituted 2-aminothiazole derivatives: Biovitrum BVT-2733, Biovitrum BVT-14225 and Biovitrum BVT-3498 [24,25,26,27] and highly selective 2-aminothiazol-4(5*H*)-es: Amgen 2922 and AMG-221 (BVT-83370) [24,28,29,30]. In the case of the 11β-HSD2 isoform, the CBX diastereoisomer (18α-glycyrrhetinic acid 3β-*O*-hemisuccinate, αCBX) is an effective inhibitor [31], whereas, among the non-steroidal inhibitors, antifungal drugs: itraconazole and posaconazole, which are the triazole derivatives, can be mentioned Scheme 2) [32]. However, due to the fact that none of these compounds has passed all the stages of clinical trials, the search for new compounds that could have therapeutic use is continued.

Our research focused on the synthesis of new thiazol-4(5*H*)-one derivatives and evaluation of their ability to inhibit 11β-HSD1 and 11β-HSD2. In addition to the inhibition of 11β-HSD1, compounds of this type show, inter alia, antiviral [33], antibacterial [34], antiparasitic [35] as well as antitumor activity [36,37,38].

## 2. Results and Discussion

### 2.1. Chemistry

In our previous work, we focused on the search for selective 11β-HSD1 inhibitors, among thiazolone and thiazolo [3,2-*α*]pyrimidin-5-one derivatives [39,40,41]. We synthesized and described 2-(allyllamino)- [39] and 2-(methylamino)-thiazol-4(5*H*)-one [40] derivatives. The analysis of the inhibitory activity of the obtained compounds in connection with the molecular modeling carried out allowed to draw conclusions as to the proper modification of the molecules in order to increase their activity and selectivity. Therefore, in the next stage of the research, we decided to focus on thiazolone derivatives containing a larger hydrophobic substituent in the amino group, which should increase the interaction of potential inhibitors with the active center of the enzyme. Now we synthesized a series of new 2-isopropylaminothiazol-4(5*H*)-one derivatives and tested their inhibitory activity against both isoforms of 11β-hydroxysteroid dehydrogenase for the purpose of determination of the selectivity of these derivatives against both 11β-HSD1 and 11β-HSD2.

New 2-(isopropylamino)thiazol-4(5*H*)-one derivatives, differing in substituents in the 5 position of the thiazole ring, were synthesized by the reaction of 1-isopropylthiourea (**1**) with the corresponding 2-bromo esters (**2a**–**2i**) (Table 1). Depending on the bromoester used, the reactions were conducted under different conditions. In the case of using aliphatic 2-bromoesters, both straight and branched, the reactions were carried out at reflux in methanol in the presence of sodium methoxide (Procedure A). Under these conditions, the five 2-(isopropylamino)thiazol-4(5*H*)-one derivatives were obtained, containing at C-5 of the thiazole ring respectively: methyl (**3a**), ethyl (**3b**), propyl (**3c**), isopropyl (**3d**) and two methyl (**3e**) groups. **3f** and **3g** derivatives with aromatic substituents at C-5 were obtained in chloroform at room temperature (Procedure B). Two compounds containing the spiro system of thiazole and cycloalkyl rings (**3h** and **3i**) were obtained by heating of the reactants in ethanol in the presence of DIPEA (*N*,*N*-diisopropylethylamine) (Procedure C).

### 2.2. In Vitro Studies

All the synthesized compounds were tested in vitro for the inhibition of 11β-hydroxysteroid dehydrogenase type 1 and type 2. The % inhibition of 11β-HSD1 and 11β-HSD2 at the inhibitor concentration of 10 µM was determined. The results are presented in Table 1.

The derivative **3h** containing a spiro system of thiazole and cyclohexane rings has the highest % inhibition of 11β-HSD1, (54.53%). It also inhibits, although to a much lesser extent, the activity of 11β-HSD2 (17.35%). In addition, the activity of 11β-HSD1 was also inhibited by the derivatives **3d**, **3e**, **3g** and **3i** (in the range of 18.06–27.58%). Comparing these values with those determined earlier for 2-(methylamino)thiazol-4(5*H*)-one derivatives [40], it turns out that they are very similar, except for the derivative **3g**. In the case of such compound with the methyl group, the activity was 10% lower. In the case of compounds **3a**–**3c** and **3f**, the inhibition of 11β-HSD1 was not observed (similarly to the methyl derivatives). This means that the increase in the volume of the substituent (replacement of the methyl group by an isopropyl group) does not significantly affect the interactions between the ligand molecule and the enzyme. Compounds that are inhibitors of 11β-HSD1, through their action, reduce the level of cortisol in the human body, which in turn translates into a reduction of the negative and long-term effects of chronic stress that accompanies people suffering from cancer. A high level of cortisol in a randomized study correlates with a lower survival of terminally ill patients [42]. Similarly, studies by Schrepf et al. showed unequivocally that in patients with ovarian cancer, women with lower cortisol levels (7.3 years on average) had a higher survival rate. For comparison, the average survival of patients with high cortisol levels was 3.3 years [43]. The above-mentioned results clearly indicate the potential use of 11β-HSD1 inhibitors in the therapy and life extension of cancer patients, especially as high serum cortisol level is an independent indicator of the survival time of terminally ill cancer patients [42].

The results of inhibition of 11β-HSD2 activity are also interesting and promising. Compounds that do not show inhibitory activity against 11β-HSD1 inhibit the activity of isoform 2 so they are selective inhibitors of this enzyme. The most active compounds were: **3c**, with the *n*-propyl group in C-5, which at a concentration of 10 µM inhibited the activity of 11β-HSD2 by more than 54%, and compound **3b** with the ethyl group in C-5 (47% inhibition of 11β-HSD2 at a concentration of 10 µM). Regulation of glucocorticoid levels is crucial for regulating cell growth. In a study by Koyama et al. on cell cultures, it was proved that the inhibition of 11β-HSD2 led to the enhancement of the antiproliferative effect of glucocorticoids [44]. Moreover, it was noticed that inhibition of 11β-HSD2 activity in mice with colorectal cancer reduces cyclooxygenase-2 (COX-2) dependent production of prostaglandin E2 (PGE2), which prevents the formation and growth of the tumor as well as its ability to metastasize. In colorectal cancer, there is a clear relationship between the production of PGE2, which comes from COX-2, and the progression of this cancer [45]. Hence, selective COX-2 inhibitors are sought. It is the glucocorticoids that induce apoptosis and are potent endogenous COX-2 inhibitors, and their action in tissues leads to the reduction of 11β-HSD2. Inhibiting the activity of this enzyme leads to an increase in the intracellular level of glucocorticoids to a level that will effectively inhibit COX-2 expression. Thus, pharmacological inhibition of 11β-HSD2 may reduce the production of PGE2 mediated by COX-2, thus preventing tumor growth and metastasis [46].

Compounds **3d** and **3e** similarly inhibit the activity of both isoforms of the enzyme. The physiological effects of glucocorticoids depend, at least in part, on inhibition of cell proliferation. Their modulation at the autocrine level is possible thanks to the 11β-HSD isoenzymes, which interconvert cortisol and inactive cortisone. Therefore, the role of 11β-HSD1 and 11β-HSD2 as autocrine elements of cell proliferation and differentiation is emphasized. Their inappropriate activity and the lack of adequate inhibition may be a factor that stimulates the growth of potentially cancerous cells [47].

### 2.3. Bioavailability

Lipiński’s rule of five (RO5) and Veber’s rule are two of the most important rules for determining the bioavailability of orally administered compounds. According to the rule of five, a compound administered orally will have good absorption and membrane penetration when it does not exceed more than one of the following parameters: molecular weight ≤ 500 Da, log *P* ≤ 5, number of hydrogen acceptors (nON) ≤ 10 and number of hydrogen donors (nOHNH) ≤ 5 [48]. On the other hand, according to the Veber’s rule, a compound will be characterized by good oral bioavailability if it meets the following two criteria: the number of rotatable bonds < 10 and the topological polar surface of a molecule (TPSA) ≤ 140 Å^2^ [49]. To initially assess bioavailability of the test compounds, the bioavailability parameters were calculated by the Molinspiration programs [50] and ALOGPS 2.1 [51] and are presented in Table 2.

The analysis of the results showed that all the obtained 2-isopropylaminothiazol-4(5*H*)-one derivatives meet the criteria of the rule of five and the Veber’s rule, which proves their good oral bioavailability.

Lipophilicity is a physicochemical parameter by which, in addition to predicting the bioavailability of a compound after oral administration, it is possible to estimate its toxicity and the ability to cross the blood-brain barrier. [52]. Log *P* values lower than 3 indicate a lower risk of toxicity of the derivatives **3a**–**3i**.

Compounds with the log *P* values in the range of 2–4, and a TPSA below 76 Å^2^ are characterized by the ability to cross the blood-brain barrier [53]. The data in Table 2 shows that all compounds have TPSA values below 76 Å^2^. While in the case of log *P* values, both of the software used indicated the derivatives **3g** and **3h** as compounds with a high chance of penetration into the central nervous system.

## 3. Materials and Methods 

### 3.1. General Informations

^1^H- and ^13^C-NMR spectra were recorded on the Bruker Avance 400 and 700 apparatus (TMS as an internal standard, Bruker Billerica, Mass., USA)

High-resolution mass spectrometry (HRMS) measurements were performed using Synapt G2-Si mass spectrometer (Waters) equipped with an ESI source and quadrupole-Time-of-flight mass analyzer. To ensure accurate mass measurements, data were collected in centroid mode and mass was corrected during acquisition using leucine enkephalin solution as an external reference (Lock-SprayTM), which generated reference ion at *m*/*z* 556.2771 Da ([M + H]^+^) in positive ESI mode. The results of the measurements were processed using the MassLynx 4.1 software (Waters, Warsaw, Poland).

### 3.2. Reagents and Solvents

All chemicals and solvents were purchased commercially and used without further purification.

*Solvents:* ethyl alcohol, methyl alcohol, chloroform, diethyl ether, ethyl acetate, dimethylsulfoxide (Avantor Performance Materials Poland S.A., Gliwice, Poland).

*Reagents for Synthesis: N*-isopropylthiourea 97% (Alfa Aesar, Kandel, Germany), ethyl 2-bromopropionate 99%, ethyl 2-bromobutyrate 98%, ethyl 2-bromovalerate 99%, ethyl 2-bromo-3-methylbutyrate 95%, ethyl 2-bromoisobutyrate 98%, ethyl 2-bromophenyl acetate 97%, ethyl 2-bromo(4-bromophenyl) acetate 97%, methyl 1-bromocyclohexane carboxylate 97%, ethyl bromocyclobutane carboxylate 95% purchased from Alfa Aesar.

*Auxiliary Reagents: N*-ethyldiisopropylamine 99% (Alfa Aesar), sodium, magnesium sulfate, hydrochloric acid, sodium hydroxide (Avantor Performance Materials Poland S.A., Gliwice, Poland)

*TLC and Column Chromatography:* 5 × 10 cm TLC plates coated with silica gel with F-254 Merck, silica gel MN kieselgel 60M with 0.04–0.063 mm grain diameter (Macherey-Nagel, Oensingen, Switzerland).

*11β-HSD1 Assays:* 18-beta-glycyrrhetinic acid (Acros Organic, Geel, Belgium), phosphate buffer powder, cortisone, NADPH tetrasodium salt (Sigma-Aldrich, Poznań, Poland), carbenoxolone (sodium salt, Cayman Chemical Company, Ann Arbor, MI, USA), Pooled human liver microsomes, mixed gender, 1 mL, 20 mg/mL Lot No.1410013, XenoTech, Cortisol Elisa Ref DkO001 Lot No. 4715A (DiaMetra, Spello, Italy), ELISA Kit for 11-Beta-Hydroxysteroid Dehydrogenase Type 1 Lot No.L160706125 (Cloud-Clone Corp., Wuhan, China), PBS Lot No. H161008 (Pan Biotech, Aidenbach, Germany).

*11β-HSD2 Assays:* 18-beta-glycyrrhetinic acid (Acros Organic), phosphate buffer powder, cortisone, NAD cofactor (Sigma-Aldrich), carbenoxolone (sodium salt, Cayman Chemical Company, Ann Arbor, MI, USA), Human Kidney Microsomes, mixed gender, 0.5 mL, 10 mg/mL Lot No. 1,710,160 XenoTech, Cortisol Elisa Ref DkO001 Lot No. 4715A (DiaMetra), Enzyme-Linked Immunosorbent Assay (ELISA) Kit for 11-Beta-Hydroxysteroid Dehydrogenase Type 2 Lot No. L191113457 (Cloud-Clone Corp.), PBS Lot No. H161008 (Pan Biotech, Aidenbach, Germany).

### 3.3. Synthesis of Compound ***3a***–***3e***—General Procedure

10 mL of methanol were measured into the flask and 0.23 g (0.01 mol) of sodium was added. After completion of the reaction, 0.59 g (0.005 mol) of *N*-isopropylthiourea (**1**) and 0.0055 mol of the corresponding 2-bromoester (**2a**–**2e**) were added. It was heated for 7–15 h depending on the type of ester (TLC control-ethyl acetate). After the heating stopped, the solvent was evaporated on a rotary evaporator and the residue was dissolved in 10 mL of water and neutralized with 2 M HCl to pH = 7–8. The product was extracted with chloroform (4 × 20 mL). The organic layer was dried with MgSO_4_, then filtered and the solvent was evaporated. The oily liquid was crystallized from diethyl ether. The resulting precipitate was filtered under reduced pressure. The NMR and MS spectra of all obtained compounds (**3a**–**3i**) are attached in Supplementary Materials [54].

2-(Isopropylamino)-5-methylthiazol-4(5*H*)-one (**3a**)—Yield: 34%. M.p. 115–117 °C. ^1^H-NMR (400 MHz, CDCl_3_, δ ppm, J Hz): 10.77 (s, 1H, N-H), 4.07 (4, 1H, C^5^-H, 7.0), 3.61 (7, 1H, CH(CH_3_)_2_, 7.0), 1.62 (d, 3H, C^5^-CH_3_, 7.0), 1.40 (dd, 6H, CH(CH_3_)_2_, 7.0 9.1). ^13^C-NMR (100 MHz, CDCl_3_, δ ppm): 188.54 (C-4), 179.79 (C-2), 48.71 (C-5), 48.61 (CH(CH_3_)_2_), 22.00 (CH_3_CH), 21.96 (CH_3_CH), 18.70 (C^5^-CH_3_). HR-MS *m*/*z* 173.0747 [M^+^ + 1] (calcd for C_7_H_13_N_2_OS: 173.0749). R_f_ (silicagel, AcOEt): 0.31.

5-Ethyl-2-(isopropylamino)thiazol-4(5*H*)-one (**3b**)—Yield: 28%. M.p. 130–131 °C. ^1^H-NMR (400 MHz, CDCl_3_, δ ppm, J Hz): 10.35 (s, 1H, N-H), 4.11 (dd, 1H, C^5^-H, 4.0 8.4), 3.67 (7, 1H, CH(CH_3_)_2_, 6.4), 2.11–2.21 (m, 1H, C^5^-CH_A_CH_3_), 1.85–1.96 (m, 1H C-5-CH_B_CH_3_), 1.43, (dd, 6H, CH(CH_3_)_2_, 5.6 6.4), 1.04 (t, 3H, C^5^-CH_2_CH_3_, 7.2). ^13^C-NMR (100 MHz, CDCl_3_, δ ppm): 187.98 (C-4), 180.55 (C-2), 56.86 (C-5), 49.04 (CH(CH_3_)_2_), 26.21 (C^5^-CH_2_CH_3_), 22.39 (2C, (CH_3_)_2_CH), 11.47 (C^5^-CH_2_CH_3_). HR-MS *m*/*z* 187.0905 [M^+^ + 1] (calcd for C_8_H_15_N_2_OS: 187.0905). R_f_ (silicagel, AcOEt): 0.38.

2-(Isopropylamino)-5-propylthiazol-4(5*H*)-one (**3c**)—Yield: 47%. M.p. 110–112 °C. ^1^H-NMR (400 MHz, CDCl_3_, δ ppm, J Hz): 10.37 (s, 1H, N-H), 4.11 (dd, 1H, C^5^-H, 4.0 9.6), 3.66 (7, 1H, CH(CH_3_)_2_, 6.4), 2.12–2.20 (m, 1H, C^5^-CH_A_CH_2_CH_3_), 1.74–1.84 (m, 1H C-5-CH_B_CH_2_CH_3_), 1.43 (dd, 6H, CH(CH_3_)_2_, 5.6 6.4), 1.45–1.55 (m, 2H, C^5^-CH_2_CH_2_CH_3_), 0.98 (t, 3H, C^5^-CH_2_CH_2_CH_3_, 7.2). ^13^C-NMR (100 MHz, CDCl_3_, δ ppm): 190.37 (C-4), 180.55 (C-2), 55.37 (C-5), 49.02 (CH(CH_3_)_2_), 35.33 (C-5-CH_2_CH_2_CH_3_), 22.34 (2C, (CH_3_)_2_CH), 21.13 (C^5^-CH_2_CH_2_CH_3_), 13.58 (C^5^-CH_2_CH_2_CH_3_). HR-MS *m*/*z* 201.1060 [M^+^ + 1] (calcd for C_9_H_17_N_2_OS: 201.1062). R_f_ (silicagel, AcOEt): 0.45.

5-Isopropyl-2-(isopropylamino)thiazol-4(5*H*)-one (**3d**)—Yield: 25%. M.p. 153–155 °C. ^1^H-NMR (400 MHz, CDCl_3_, δ ppm, J Hz): 10.54 (s, 1H, N-H), 4.20 (d, 1H, C^5^-H, 3.6), 3.71 (7, 1H, CH(CH_3_)_2_, 6.8), 2.52–2.63 (m, 1H, C^5^-CH), 1.43 (dd, 6H, CH(CH_3_)_2_, 6.0 6.4), 1.07 (d, 3H, C^5^-CHCH_3A_, 6.8), 0.91 (d, 3H, C^5^-CHCH_3B_, 6.8). ^13^C-NMR (100 MHz, CDCl_3_, δ ppm): 187.70 (C-4), 180.83 (C-2), 62.95 (C-5), 49.04 (CH(CH_3_)_2_), 30.53 (C^5^-CH), 22.33 (2C, (CH_3_)_2_CH), 16.30 (2C, C^5^-CH(CH_3_)_2_). HR-MS *m*/*z* 201.1062 [M^+^ + 1] (calcd for C_9_H_17_N_2_OS: 201.1062). R_f_ (silicagel, AcOEt): 0.43.

2-(Isopropylamino)-5,5-dimethylthiazol-4(5*H*)-one (**3e**)—Yield: 10%. M.p. 193–194 °C. ^1^H-NMR (400 MHz, CDCl_3_, δ ppm, J Hz): 10.43 (s, 1H, N-H), 3.59 (7, 1H, CH(CH_3_)_2_, 6.8), 1.64 (s, 6H, C^5^(CH_3_)_2_), 1.42 (d, 6H, CH(CH_3_)_2_, 6.8).m ^13^C-NMR (100 MHz, CDCl_3_, δ ppm): 193.09 (C-4), 178.76 (C-2), 59.84 (C-5), 49.04 (CH(CH_3_)_2_), 27.92 (2C, C^5^-(CH_3_)_2_) 22.46 (2C, (CH_3_)_2_CH). HR-MS *m*/*z* 187.0907 [M^+^ + 1] (calcd for C_8_H_15_N_2_OS: 187.0905). R_f_ (silicagel, AcOEt): 0.44.

### 3.4. Synthesis of Compound ***3f***,***g***—General Procedure

1.18 g (0.01 mol) of *N*-isopropylthiourea (**1**) and 0.011 mol of the corresponding 2-bromoester (**2f**,**g**) were added to 50 mL of chloroform. The mixture was stirred at room temperature for 168–240 h (TLC control-ethyl acetate). The resulting crude product was filtered off under reduced pressure and crystallized from ethyl acetate [39].

2-(Isopropylamino)-5-phenylthiazol-4(5*H*)-one (**3f**)—Yield: 25%. M.p. 227 °C (dec.). ^1^H-NMR (400 MHz, CDCl_3_, δ ppm, J Hz): 12.07 (s, 1H, N-H), 7.44–7.49 (m, 3H, C_6_H_5_), 7.37–7.41 (m, 2H, C_6_H_5_), 5.50 (s, 1H, C^5^-H), 3.80 (7, 1H, CH(CH_3_)_2_, 6.0), 1.54 (d, 6H, CH(CH_3_)_2_, 6.0). ^13^C-NMR (100 MHz, DMSO, δ ppm): 171.62 (C-4), 169.95 (C-2), 131.25 (1C, C_6_H_5_), 130.25 (1C, C_6_H_5_), 129.79 (2C, C_6_H_5_), 128.67 (2C, C_6_H_5_), 53.85 (C-5), 51.72 (CH(CH_3_)_2_), 21.89 (1C, (CH_3_)_2_CH). 21.80 (1C, (CH_3_)_2_CH). HR-MS *m*/*z* 235.0909 [M^+^ + 1] (calcd for C_12_H_15_N_2_OS: 235.0905). R_f_ (silicagel, AcOEt): 0.60.

5-(4-Bromophenyl)-2-(isopropylamino)thiazol-4(5*H*)-one (**3g**)—Yield: 85%. M.p. 236–237 °C. ^1^H-NMR (400 MHz, DMSO, δ ppm, J Hz): 9.72 (s, 1H, N-H), 7.54–7.59 (m, 2H, C_6_H_4_), 7.24–7.32 (m, 2H, C_6_H_4_), 5.49 (s, 1H, C^5^-H), 3.75 (7, 1H, CH(CH_3_)_2_, 1.84), 1.22 (dd, 6H, CH(CH_3_)_2_, 6.6 9.0). ^13^C-NMR (100 MHz, DMSO, δ ppm): 185.47 (C-4), 176.68 (C-2), 137.04 (1C, C_6_H_5_), 132.05 (2C, C_6_H_5_), 131.10 (2C, C_6_H_5_), 121.60 (1C, C_6_H_5_), 57.03 (C-5), 48.25 (CH(CH_3_)_2_), 22.30 (1C, (CH_3_)_2_CH). 22.27 (1C, (CH_3_)_2_CH). HR-MS *m*/*z* 313.0011 [M^+^ + 1] (calcd for C_12_H_14_N_2_OS^79^Br: 313.0010). R_f_ (silicagel, AcOEt): 0.60.

### 3.5. Synthesis of Compound ***3h***,***i***—General Procedure

2.025 mL (0.011 mol) *N*,*N*-diisopropylethylamine, 1.18 g (0.01 mol) *N*-isopropylthiourea (**1**) and 0.01 mol of the corresponding 2-bromoester (**2h**,**i**) were added to 3.5 mL of ethyl alcohol. It was heated to reflux for 168–240 h (TLC control-ethyl acetate). After the end of heating, the solvent was evaporated and the residue was dissolved in 10 mL of water, the pH of the solution was about 7. The product was extracted with chloroform (4 × 20 mL). The organic layer was dried with anhydrous MgSO_4_. Then it was filtered off and the solvent was evaporated. The reaction mixture was purified by column chromatography [39].

2-(Isopropylamino)-1-thia-3-azaspiro [4,5]dec-2-en-4-one (**3h**)—Yield: 8%. M.p. 140 °C (dec.). ^1^H-NMR (400 MHz, CDCl_3_, δ ppm, J Hz): 5.63 (s, 1H, N-H), 3.68 (7, 1H, CH(CH_3_)_2_, 6.8), 1.81–2.16 (m, 6H, C_5_H_10_), 1.43 (d, 6H, CH(CH_3_)_2_, 6.8), 1.11–1.32 (m, 4H, C_5_H_10_). ^13^C-NMR (100 MHz, CDCl_3_, δ ppm): 189.93 (C-4), 179.18 (C-2), 68.82 (C-5), 49.21 (CH(CH_3_)_2_), 36.59 (2C, C_5_H_10_), 25.32 (2C, C_5_H_10_), 24.90 (1C, C_5_H_10_), 22.36 (2C, (CH_3_)_2_CH). HR-MS *m*/*z* 227.1221 [M^+^ + 1] (calcd for C_11_H_19_N_2_OS: 227.1218). R_f_ (silicagel, AcOEt): 0.56.

2-(Isopropylamino)-1-thia-3-azaspiro [3,4]oct-2-en-4-one (**3i**)—Yield: 15%. M.p. 151–152 °C. ^1^H-NMR (400 MHz, CDCl_3_, δ ppm, J Hz): 10.15 (s, 1H, N-H), 3.58 (7, 1H, CH(CH_3_)_2_, 6.4), 2.73–2.87 (m, 2H, C_3_H_6_), 2.46–2.59 (m, 2H, C_3_H_6_), 2.26–2.39 (m, 1H, C_3_H_6_), 1.97–2.10 (m, 1H, C_3_H_6_), 1.44 (d, 6H, CH(CH_3_)_2_, 6.4). ^13^C-NMR (100 MHz, CDCl_3_, δ ppm): 190.84 (C-4), 178.76 (C-2), 60.48 (C-5), 49.08 (CH(CH_3_)_2_), 34.17 (2C, C_3_H_6_), 22.40 (2C, (CH_3_)_2_CH), 16.89 (1C, C_3_H_6_). HR-MS *m*/*z* 199.0910 [M^+^ + 1] (calcd for C_9_H_15_N_2_OS: 199.0905). R_f_ (silicagel, AcOEt): 0.48.

### 3.6. Inhibition of 11β-HSD1 Assays

Transformation of cortisone to cortisol was conducted on 96-well microtiter plates in the presence of the human liver microsomes as 11β-HSD1 source in a total volume of 100 μL. 20 μL of mixture cortisone/NADPH (final concentration 200 nM/2 μM), 10 μL of microsomes (1.13 μg/mL 11β-HSD1) solution in PBS (final quantity 2.5 μg), 60 μL of phosphate buffer (pH 7.4) and 10 μL of inhibitor solution (solvent DMSO/water 1/99, final concentration 10 μM) were placed in the well. Mixtures were incubated for 150 min at 37 °C. The reaction was stopped by the addition of 10 μL solution of 100 μM 18β-glycyrrhetinic acid in PBS. The amount of cortisol obtained was measured using a cortisol ELISA kit (Diametra, Via Pozzouolo, Spello, Peruga, Italy) [11].

### 3.7. Inhibition of 11β-HSD2 Assays

Transformation of cortisol to cortisone was conducted on 96-well microtiter plates in the presence of human kidney microsomes as 11β-HSD2 sources in a total volume of 100 μL. 20 μL of substrate mixture cortisol/NAD^+^ (final concentration 200 nM/2 μM), 10 μL of microsomes (0.127 μg/mL 11β-HSD2) solution in PBS (final quantity 2.5 μg), 60 μL of phosphate buffer (pH 7.4) and 10 μL of inhibitor solution (solvent DMSO/water 1/99, final concentration 10 μM) were placed in the well. Mixtures were incubated for 150 min at 37 °C. The reaction was stopped by the addition of 10 μL solution containing 100 μM carbenoxolone in PBS. The amount of unreacted cortisol was measured using a cortisol ELISA kit [11].

### 3.8. Determination of IC_50_

IC_50_ values were determined by carrying out analogous determinations as those described in point 3.6 (for 11β-HSD1 inhibitors) or in point 3.7 (for 11β-HSD2 inhibitors) using inhibitors at the following concentrations: 10, 5, 2.5, 1.25, 0.625 µM. Then, graphs of the dependence of the concentration of cortisol (in the case of 11β-HSD1 inhibitors) and cortisone (in the case of 11β-HSD2 inhibitors) obtained in reaction to the concentration of the inhibitor were prepared. The inhibitor concentration at which there was a 50% reduction of cortisol (or cortisone) concentration compared to the blank (reaction without inhibitor) was read from the graph.

## 4. Conclusions

In conclusion, we described the synthesis of new 2-(isopropylamino)thiazol-4(5*H*)-one derivatives and examined their inhibitory activity against isoforms 1 and 2 of 11β-HSD. The derivative **3h**, containing the spiro system of thiazole and cyclohexane rings in its structure, is characterized by the highest degree of 11β-HSD1 inhibition (54.53% at the concentration 10 µM) and is the most selective inhibitor of this enzyme among the tested compounds. In turn, derivatives **3b** and **3c** inhibit the activity of 11β-HSD2 to a high degree (47.08 and 54.59% respectively at the concentration 10 µM) and are completely selective. Therefore, they can potentially contribute to a reduction in PGE2 production, thereby preventing the proliferation of tumor cells, tumor growth, and metastasis in, for example, colorectal cancer. The obtained results may constitute a new therapeutic approach targeting the action of 11β-HSD1 and 11β-HSD2 in order to restore the correct response to the action of glucocorticoids, which may be important in the inhibition of the proliferation of neoplastic cells.

## Figures and Tables

**Table 1:**
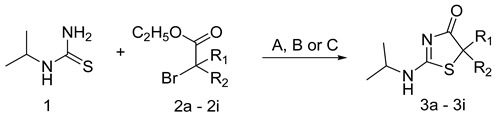
Synthesis and inhibitory activity of 2-(isopropylamino)thiazol-4(5*H*)-one derivatives.

No	R_1_	R_2_	Procedure Time (h)	Isolated Yield (%)	M.p. (°C)	% of 11β-HSD1 Inhibition 10 μM/IC_50_ [µM] ^a^	% of 11β-HSD2 Inhibition 10 μM/IC_50_ [µM] ^a^
**3a**	H	CH_3_	A/7	34	115–117	0	27.61 ± 2.11/nd
**3b**	H	C_2_H_5_	A/8	28	130–131	0	47.08 ± 2.51/nd
**3c**	H	*n*-C_3_H_7_	A/10	47	110–112	0	54.59 ± 4.21/9.12 ± 0.76
**3d**	H	CH(CH_3_)_2_	A/15	25	153–155	18.86 ± 3.47/nd	14.29 ± 1.21/nd
**3e**	CH_3_	CH_3_	A/11	10	193–194	18.06 ± 0.97/nd	21.43 ± 2.11/nd
**3f**	H	C_6_H_5_	B/240	25	227 (dec.)	0	10.48 ± 2.13/nd
**3g**	H	*p*Br-C_6_H_4_	B/168	85	236–237	27.58 ± 2.53/nd	27.61 ± 2.11/nd
**3h**	C_5_H_10cycl_		C/168	8	140 (dec.)	54.53 ± 3.03/9.35 ± 0.67	17.35 ± 1.25/nd
**3i**	C_3_H_6cycl_		C/240	15	151–152	20.94 ± 2.22/nd	36.73 ± 2.43/nd
**control**						84.54 ± 5.47 ^b^/<0.625	47.43 ± 1.11 ^c^/nd

Procedure A: MeOH, MeONa; reflux, Procedure B: CHCl_3_, RT; Procedure C: EtOH, DIPEA, reflux, nd —Not determined due to too low % enzyme inhibition value (the cutoff value for this assay was 50% inhibition at the inhibitor concentration of 10 µM), ^a^ values were obtained from three independent determinations, IC_50_ were determined with 5 inhibitor concentrations, ^b^ for carbenoxolone, ^c^ for 18β-glycyrrhetinic acid.

**Table 2:** Bioavailability parameters of 2-(isopropylamino)thiazol-4(5*H*)-one derivatives calculated with the Molinspiration and ALOGPS 2.1 software.

No.	Lipinski’s Rule of Five					Veber’s Rule	
	Molecular Weight ^a^	miLog *P* ^a^	Log *P* ^b^	nOHNH ^a^	nON ^a^	TPSA/Å^2 a^	Nrotb ^a^
**3a**	172.25	0.75	1.01	1	3	41.46	2
**3b**	186.28	1.25	1.86	1	3	41.46	3
**3c**	200.31	1.81	2.37	1	3	41.46	4
**3d**	200.31	1.50	1.53	1	3	41.46	3
**3e**	186.28	1.20	1.39	1	3	41.46	2
**3f**	234.32	1.97	2.26	1	3	41.46	3
**3g**	313.22	2.78	2.82	1	3	41.46	3
**3h**	226.34	2.37	2.54	1	3	41.46	2
**3i**	198.29	1.12	1.88	1	3	41.46	2

^a^ Molinspiration; ^b^ ALOGPS 2.1.

## Supplementary Materials

The following are available online at https://www.mdpi.com/1420-3049/25/18/4233/s1, Figure S1.1: ^1^H NMR spectra of compound **3a**, Figure S1.2: ^1^H NMR spectra of compound **3b**, Figure S1.3: ^1^H NMR spectra of compound **3c**, Figure S1.4: ^1^H NMR spectra of compound **3d**, Figure S1.5: ^1^H NMR spectra of compound **3e**, Figure S1.6: ^1^H NMR spectra of compound **3f**, Figure S1.7: ^1^H NMR spectra of compound **3g**, Figure S1.8: ^1^H NMR spectra of compound **3h**, Figure S1.9: ^1^H NMR spectra of compound **3i**, Figure S2.1: ^13^C NMR spectra of compounds **3a**, Figure S2.2: ^13^C NMR spectra of compounds **3b**, Figure S2.3: ^13^C NMR spectra of compounds **3c**, Figure S2.4: ^13^C NMR spectra of compounds **3d**, Figure S2.5: ^13^C NMR spectra of compounds **3e**, Figure S2.6: ^13^C NMR spectra of compounds **3f**, Figure S2.7: ^13^C NMR spectra of compounds **3g**, Figure S2.8: ^13^C NMR spectra of compounds **3h**, Figure S2.9: ^13^C NMR spectra of compounds **3i**, Figure S3.1: HRMS spectra of compounds **3a**, Figure S3.2: HRMS spectra of compounds **3b**, Figure S3.3: HRMS spectra of compounds **3c**, Figure S3.4: HRMS spectra of compounds **3d**, Figure S3.5: HRMS spectra of compounds **3e**, Figure S3.6: HRMS spectra of compounds **3f**, Figure S3.7: HRMS spectra of compounds **3g**, Figure S3.8: HRMS spectra of compounds **3h**, Figure S3.9: HRMS spectra of compounds **3i**.
