# Supplementary material for: Synthesis of Novel 2-(Isopropylamino)thiazol-4(5H)-one Derivatives and Their Inhibitory Activity of 11β-HSD1 and 11β-HSD2 in Aspect of Carcinogenesis Prevention
Source: Molecules. 2020 Sep 15;25(18):4233. doi: 10.3390/molecules25184233 (PMC7570983; doi:10.3390/molecules25184233)
Supplement: Supplementary file 1 [file molecules-25-04233-s001.pdf]

Supplementary information

Synthesis of novel 2-(isopropylamino)thiazol-4(5*H*)-one derivatives and their inhibitory activity of 11β-HSD1 and 11β-HSD2 in aspect of carcinogenesis prevention.

Daria Kupczyk [1,*,#], Renata Studzińska [2,*,#], Rafał Bilski [1], Szymon Baumgart [2], Renata Kołodziejska [1] and Alina Woźniak[1]

[1] Nicolaus Copernicus University in Toruń, Collegium Medicum in Bydgoszcz, Faculty of Medicine, Department of Medical Biology and Biochemistry, Karłowicza 24, 85–092 Bydgoszcz, Poland; dariak@cm.umk.pl
[2] Nicolaus Copernicus University in Toruń, Collegium Medicum in Bydgoszcz, Faculty of Pharmacy, Department of Organic Chemistry, Jurasza 2, 85–089 Bydgoszcz, Poland; rstud@cm.umk.pl
* Correspondence: dariak@cm.umk.pl (D.K.); rstud@cm.umk.pl (R.S.)

[#] Renata Studzińska and Daria Kupczyk have equally contributed to the present paper and should be considered to be the first authors

S1. ^{1}H NMR spectra of compounds **3a** – **3i**

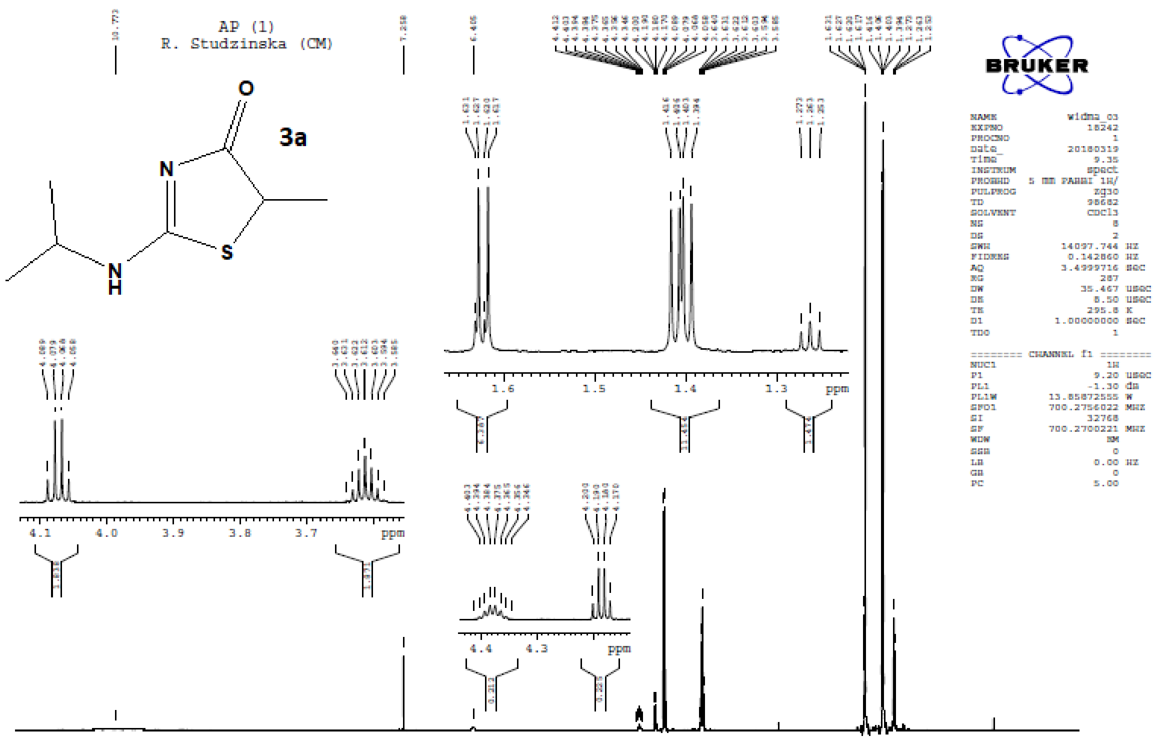

S1.1. ^{1}H NMR spectra of compound **3a**

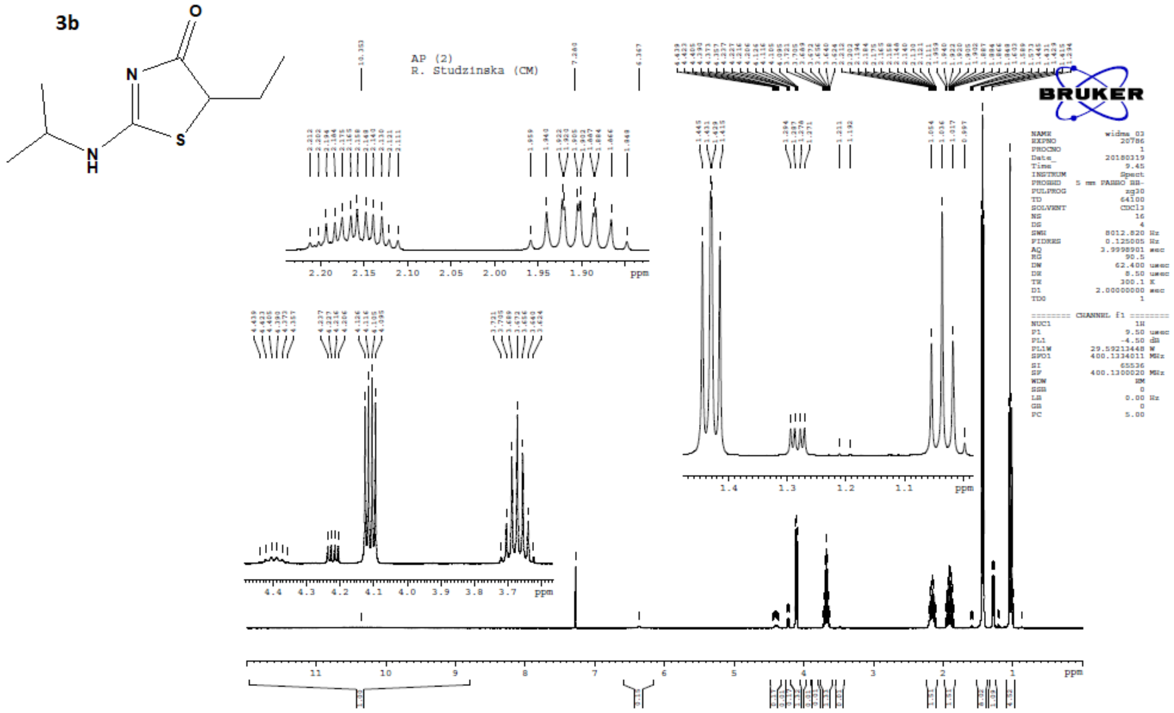

S1.2. ^{1}H NMR spectra of compound **3b**

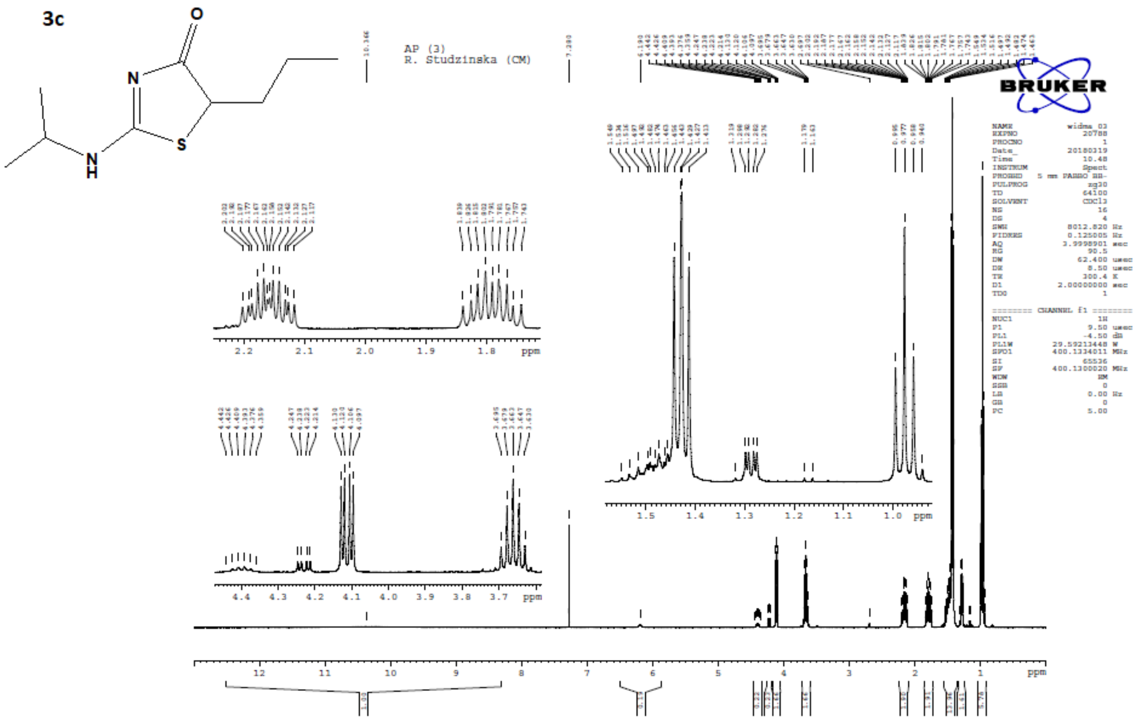

S1.3. ^{1}H NMR spectra of compound **3c**

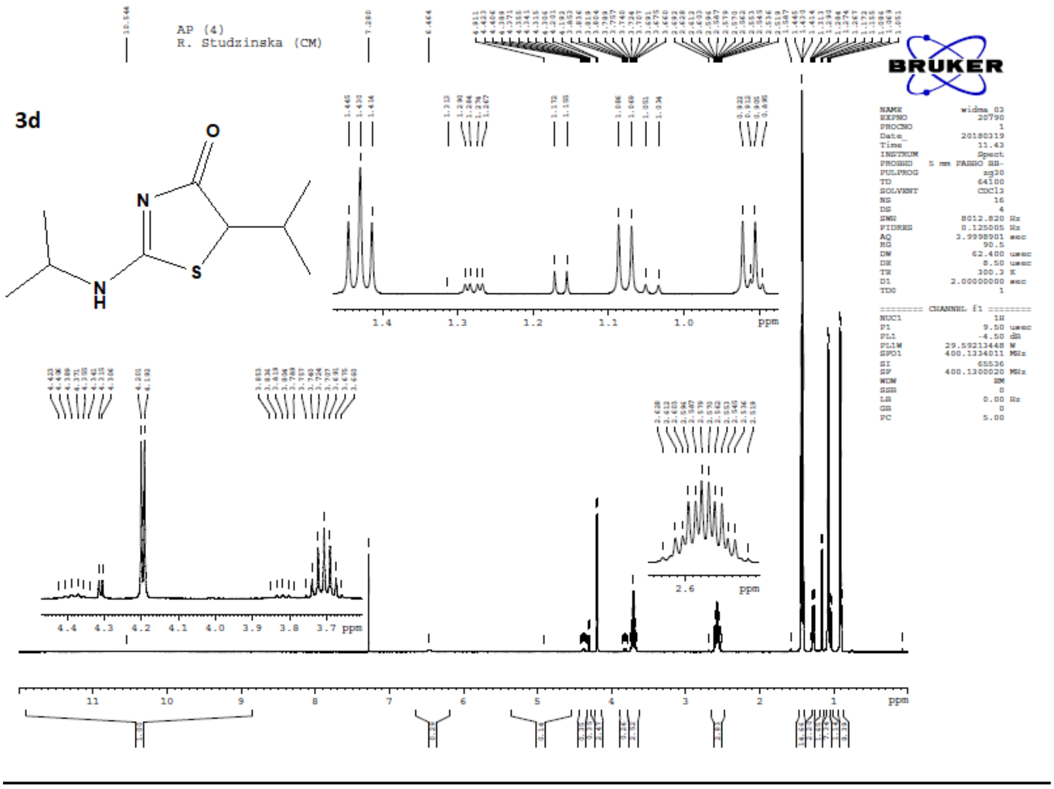

S1.4. ^{1}H NMR spectra of compound **3d**

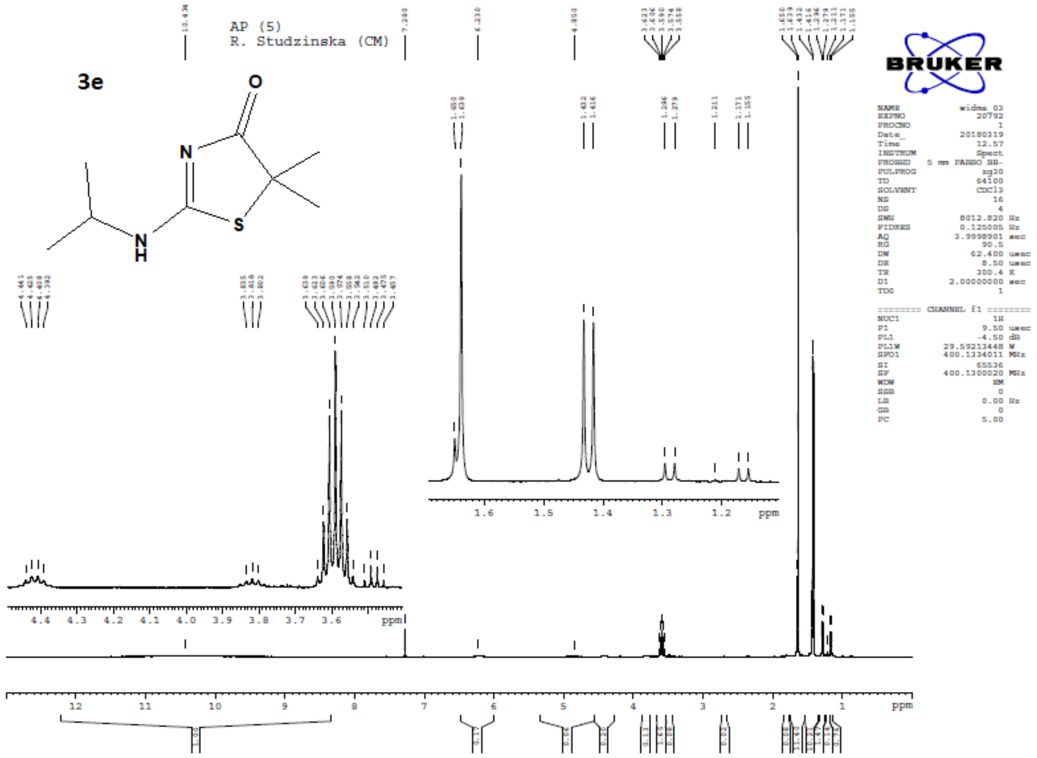

S1.5. ^{1}H NMR spectra of compound **3e**

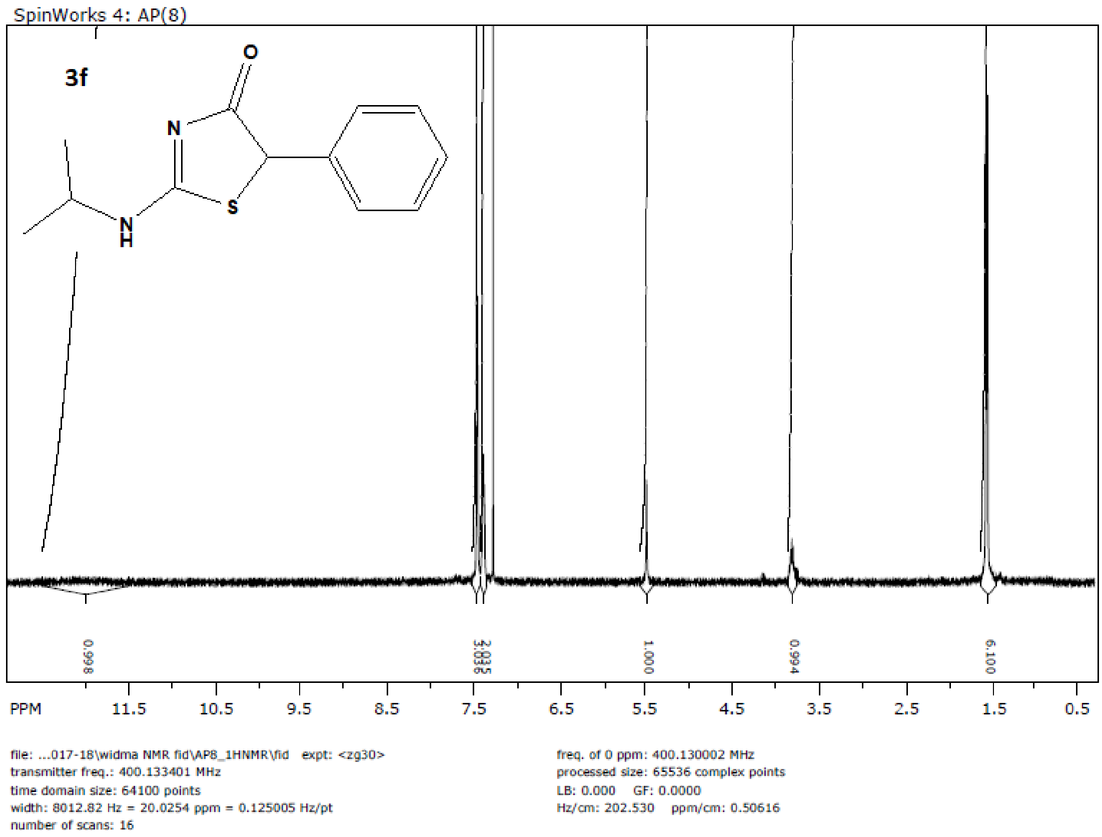

S1.6. ^{1}H NMR spectra of compound **3f**

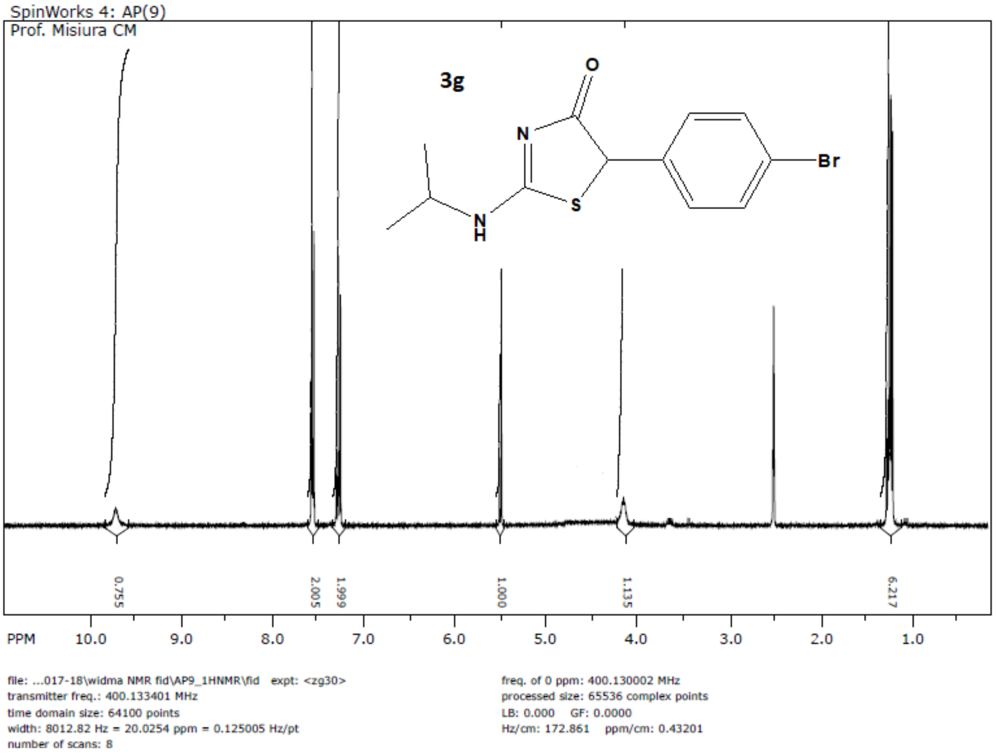

S1.7. ^{1}H NMR spectra of compound **3g**

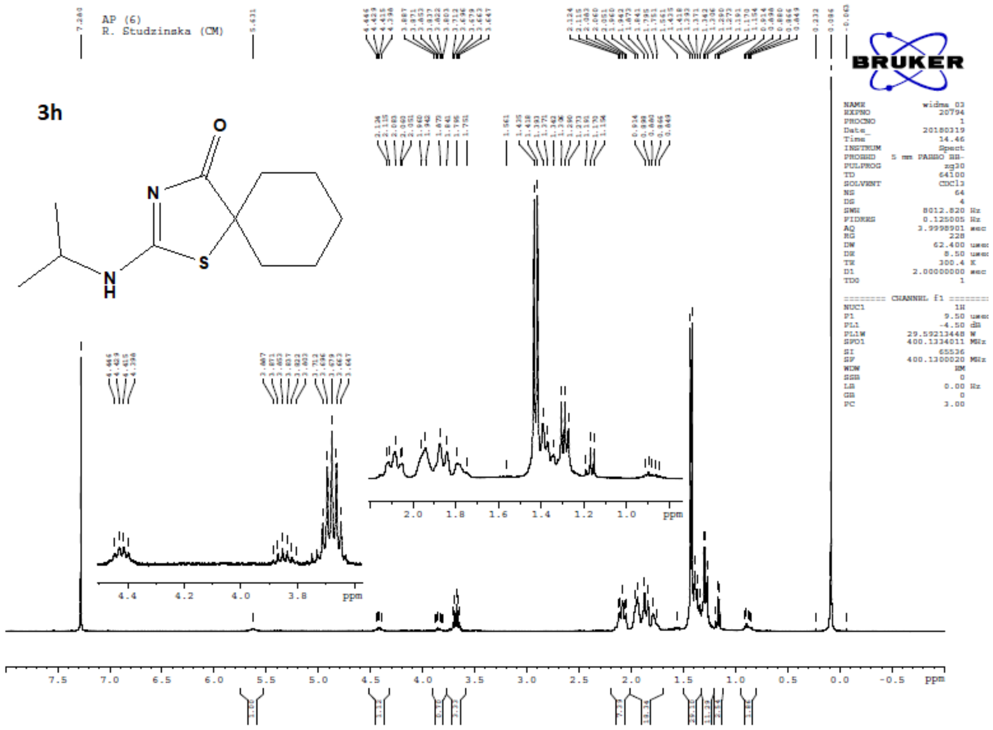

S1.8. ^{1}H NMR spectra of compound **3h**

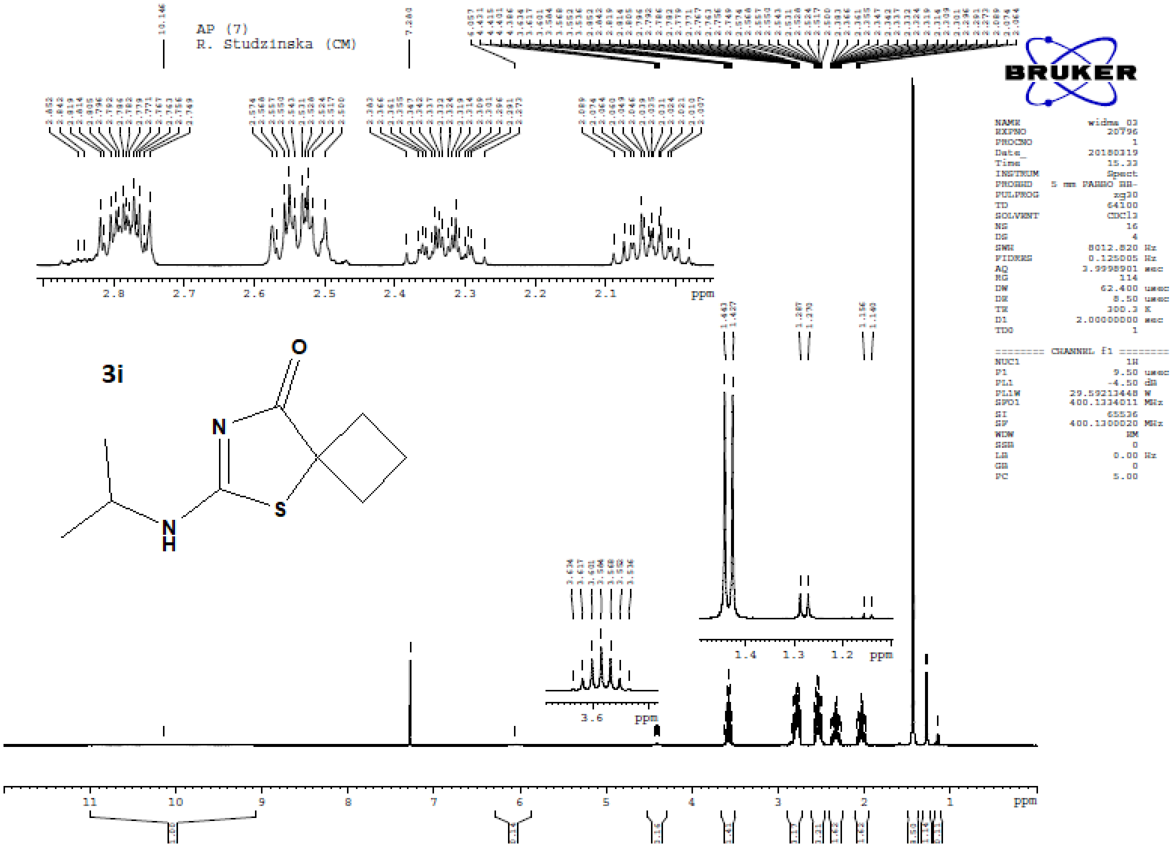

S1.9. ¹H NMR spectra of compound **3i**

S2. ^{13}C NMR spectra of compounds **3a – 3i**

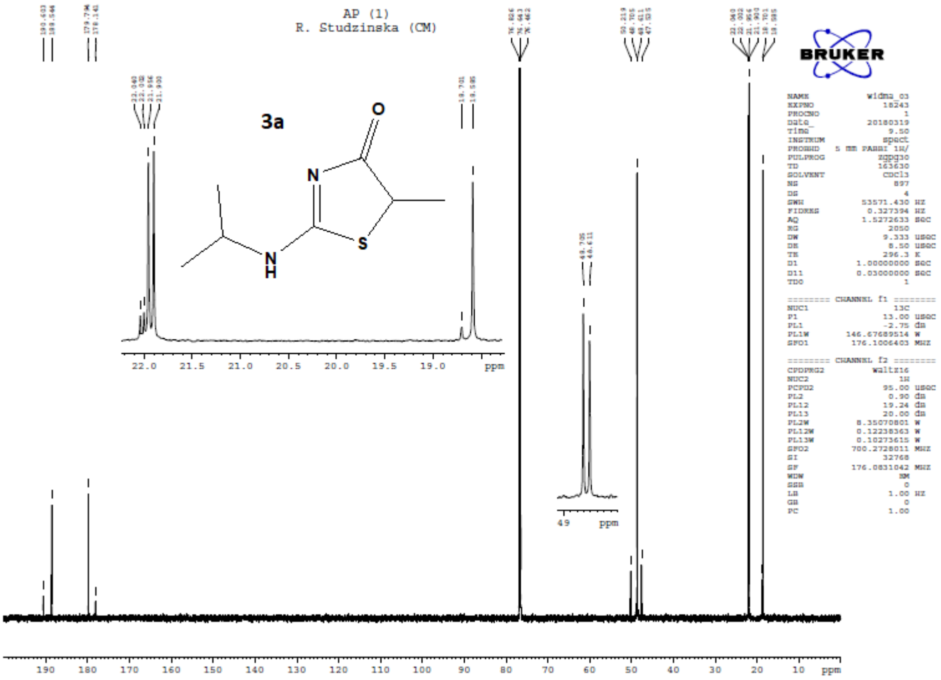

S2.1. ^{13}C NMR spectra of compounds **3a**

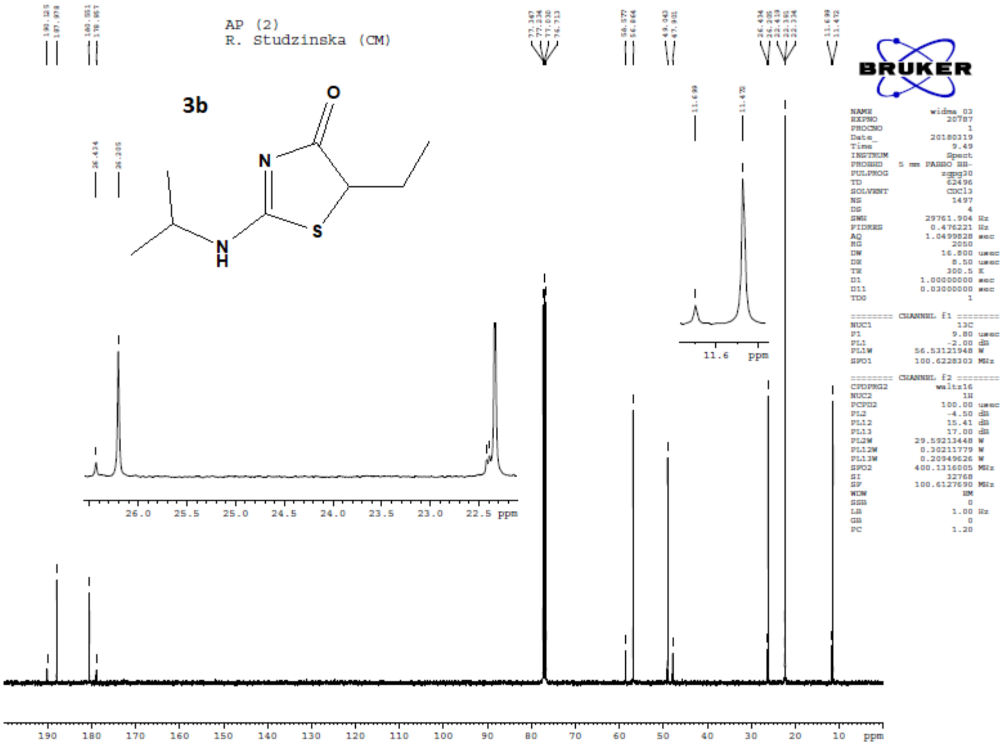

S2.2. ^{13}C NMR spectra of compounds **3b**

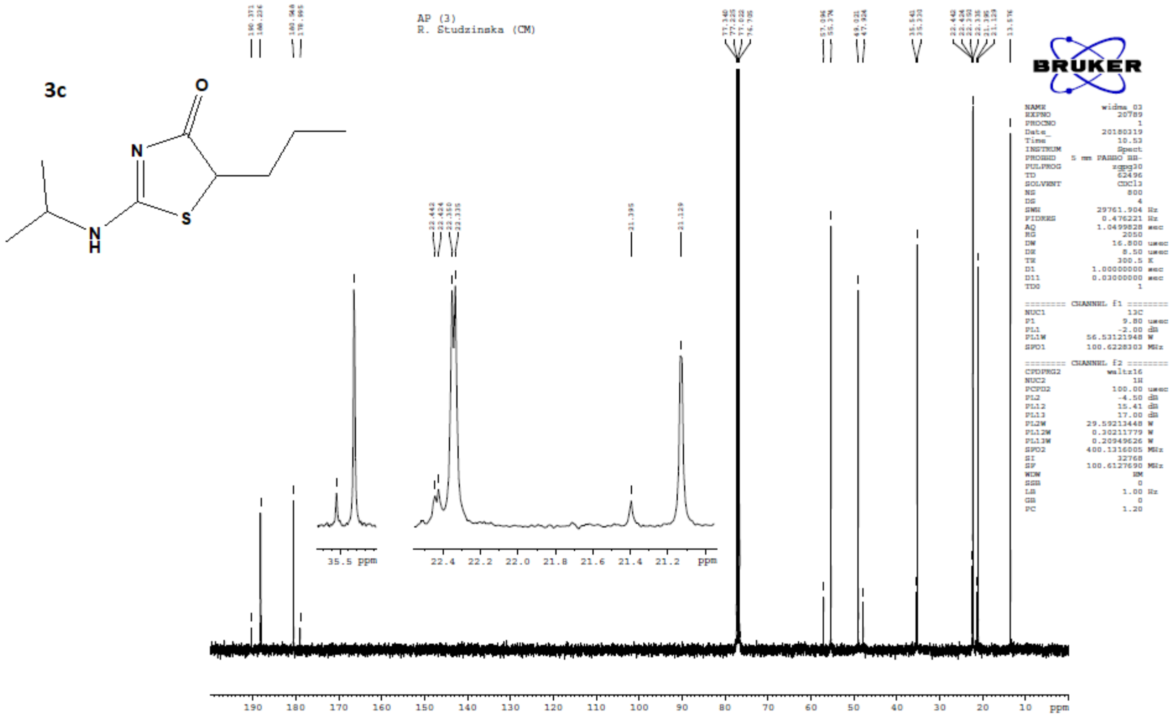

S2.3. ^{13}C NMR spectra of compounds **3c**

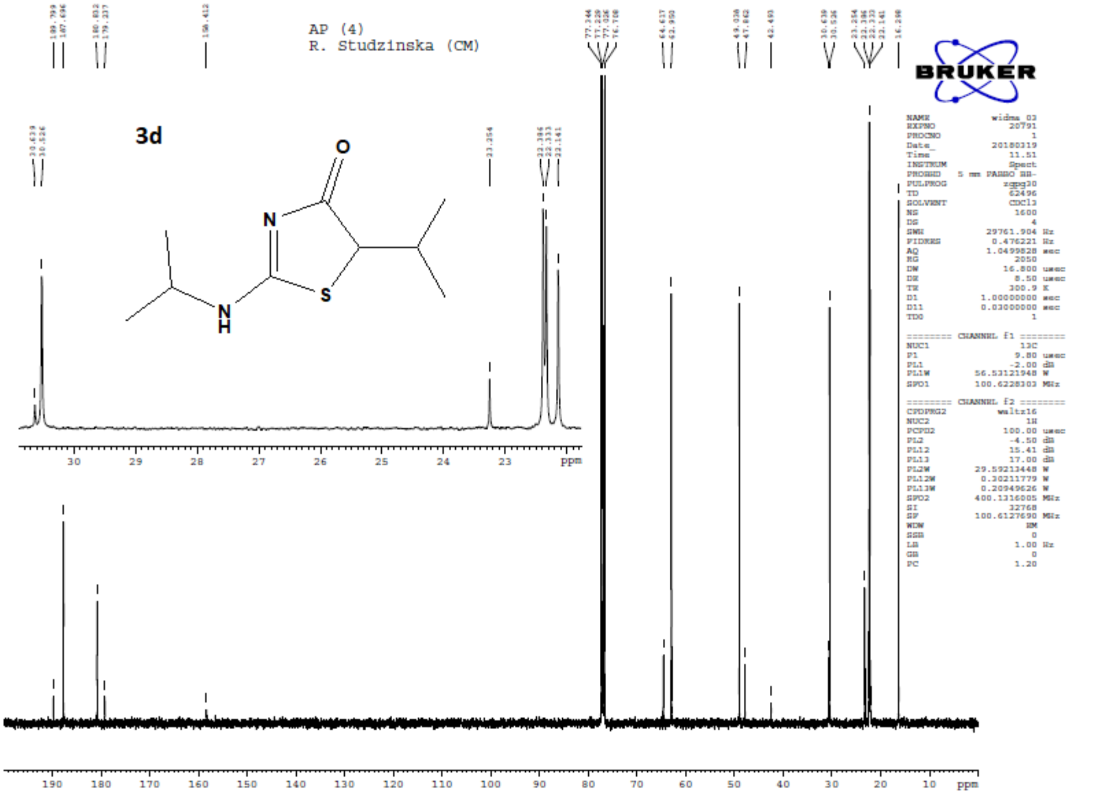

S2.4. ^{13}C NMR spectra of compounds **3d**

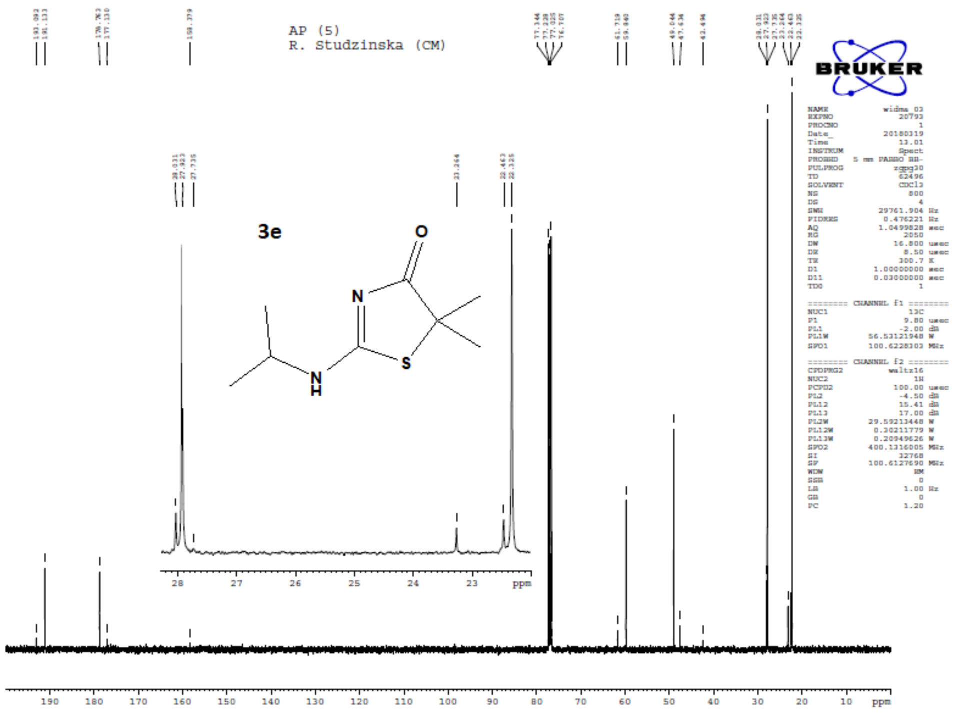

S2.5. ^{13}C NMR spectra of compounds **3e**

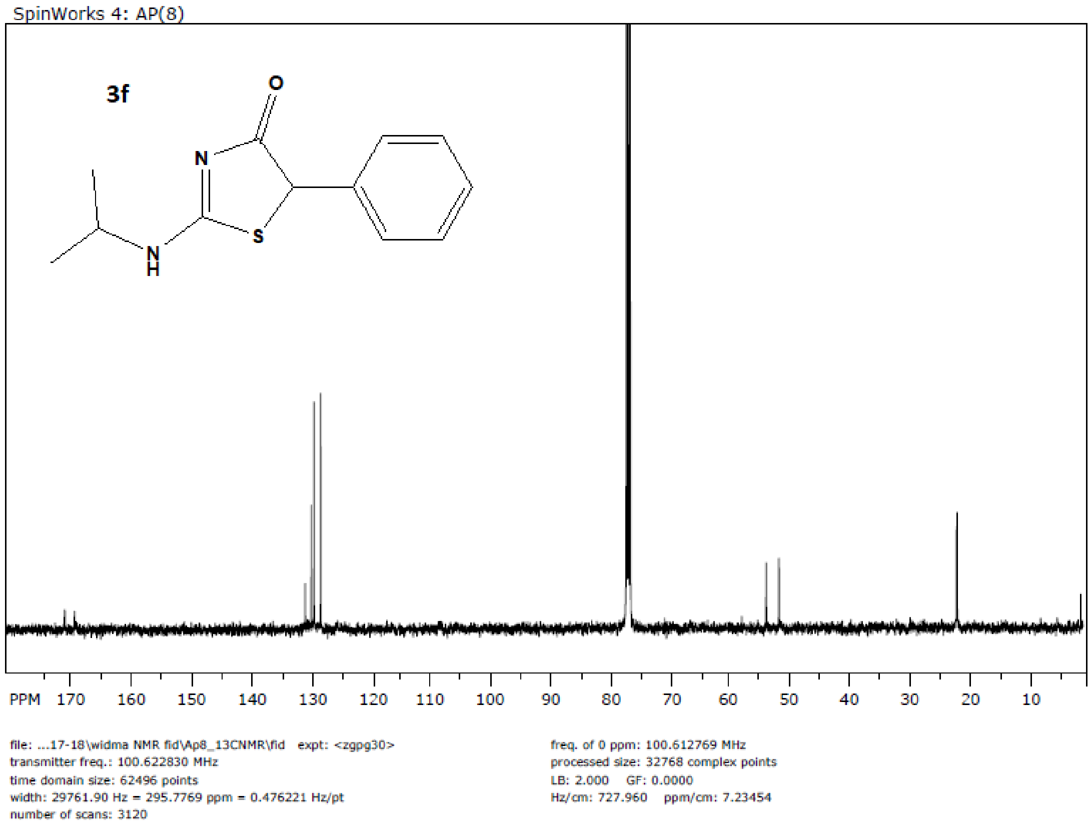

S2.6. ^{13}C NMR spectra of compounds **3f**

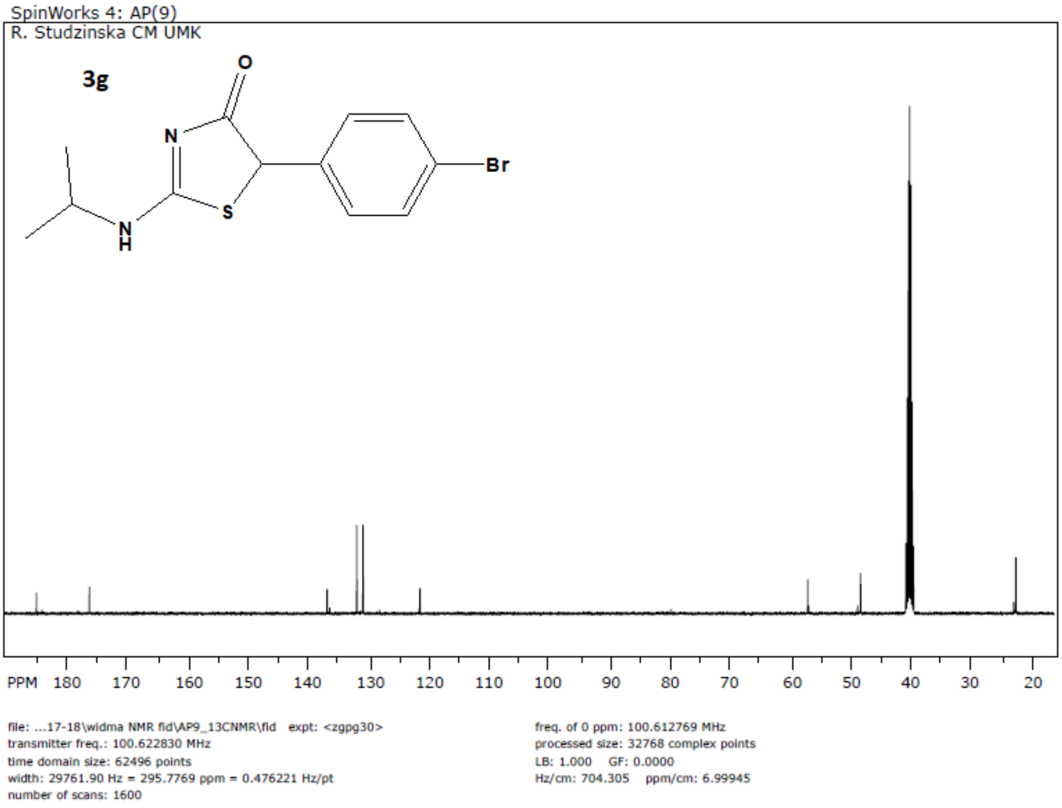

S2.7. ^{13}C NMR spectra of compounds **3g**

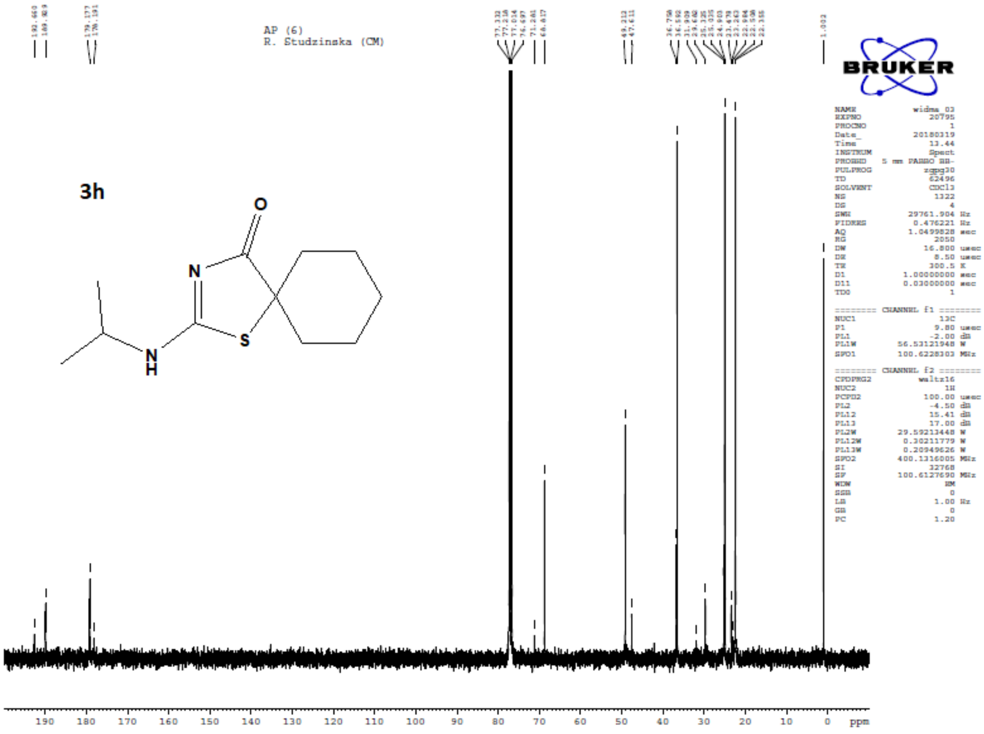

S2.8. ^{13}C NMR spectra of compounds **3h**

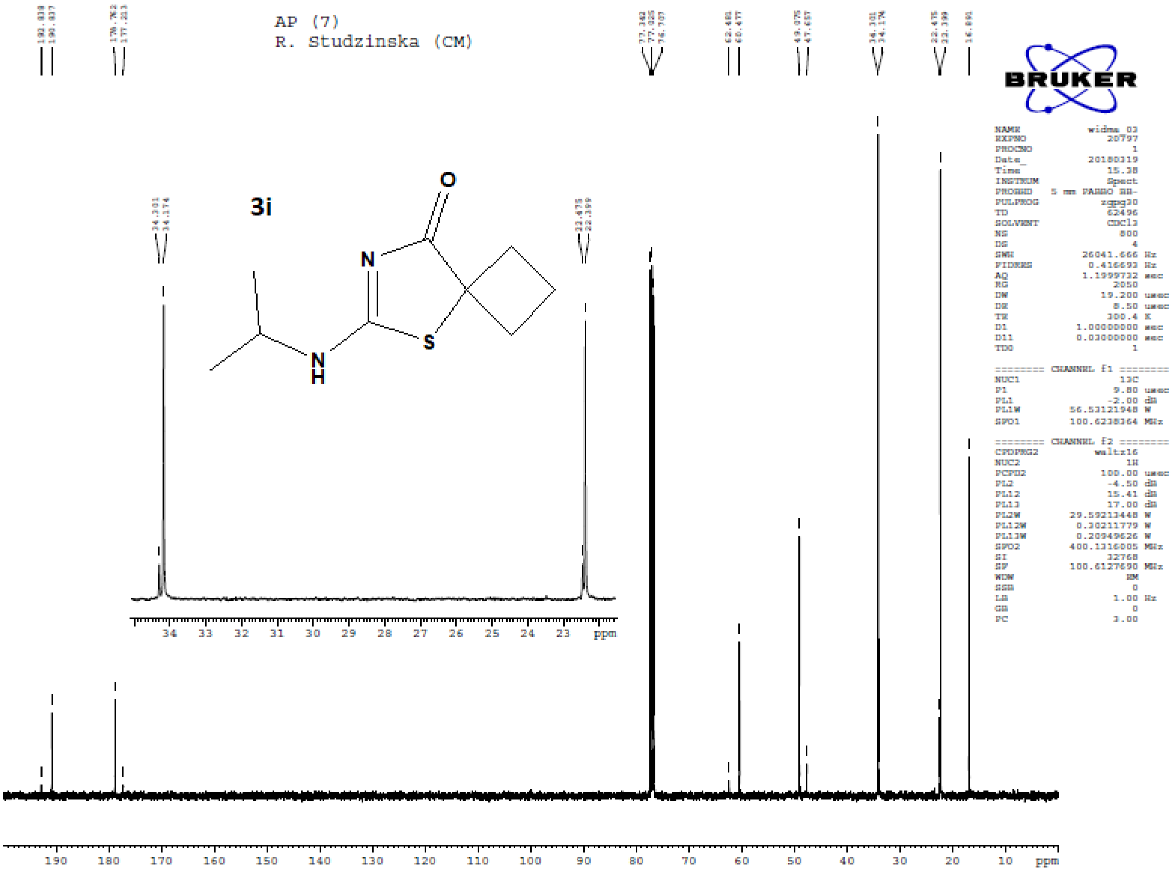

S2.9. ^{13}C NMR spectra of compounds **3i**

S3. HRMS spectra of compounds **3a** – **3i**

Elemental Composition Report **Page 1**

3a

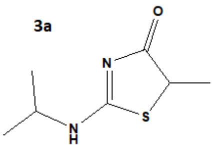

Single Mass Analysis
Tolerance = 10.0 PPM / DBE: min = -1.5, max = 50.0
Element prediction: Off
Number of isotope peaks used for i-FIT = 3

Monoisotopic Mass, Even Electron Ions
62 formula(e) evaluated with 1 results within limits (up to 50 closest results for each mass)
Elements Used:
C: 0-12 H: 0-20 N: 0-3 O: 0-2 S: 0-2
R.Studzinska
180515_AP_1 7 (0.194) Cm (7:8-16:29)

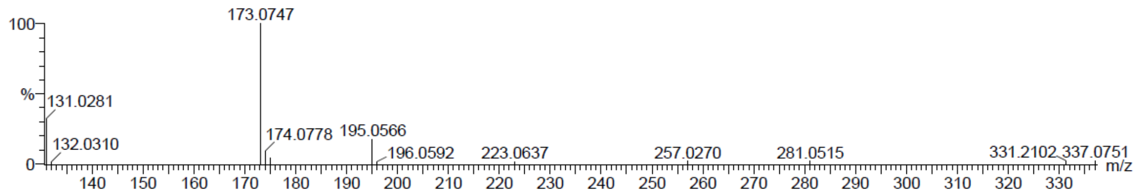

Minimum:				-1.5				
Maximum:		15.0	10.0	50.0				
Mass	Calc. Mass	mDa	PPM	DBE	i-FIT	Norm	Conf(%)	Formula
173.0747	173.0749	-0.2	-1.2	2.5	27.1	n/a	n/a	C7 H13 N2 O S

S3.1. HRMS spectra of compounds **3a**

Elemental Composition Report **Page 1**

3b

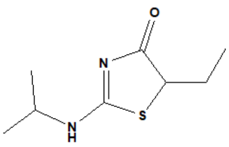

Single Mass Analysis
Tolerance = 10.0 PPM / DBE: min = -1.5, max = 50.0
Element prediction: Off
Number of isotope peaks used for i-FIT = 3

Monoisotopic Mass, Even Electron Ions
52 formula(e) evaluated with 1 results within limits (up to 50 closest results for each mass)
Elements Used:
C: 0-10 H: 0-20 N: 0-3 O: 0-2 S: 0-2
R.Studzinska
180515_AP_2 7 (0.194) Cm (7:9-18:29)

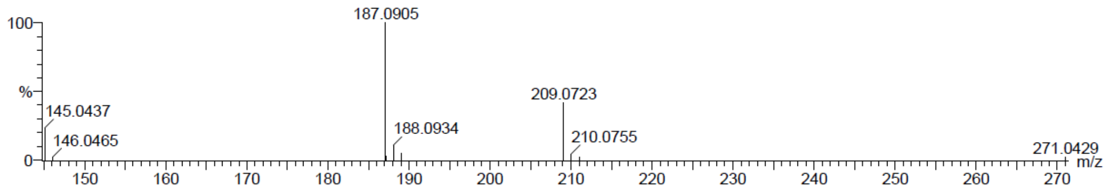

Minimum:				-1.5				
Maximum:		15.0	10.0	50.0				
Mass	Calc. Mass	mDa	PPM	DBE	i-FIT	Norm	Conf(%)	Formula
187.0905	187.0905	0.0	0.0	2.5	54.1	n/a	n/a	C8 H15 N2 O S

S3.2. HRMS spectra of compounds **3b**

Elemental Composition Report

3c

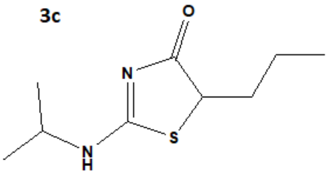

Single Mass Analysis

Tolerance = 10.0 PPM / DBE: min = -1.5, max = 50.0
Element prediction: Off
Number of isotope peaks used for i-FIT = 3

Monoisotopic Mass, Even Electron Ions
65 formula(e) evaluated with 1 results within limits (up to 50 closest results for each mass)
Elements Used:
C: 0-15 H: 0-20 N: 0-3 O: 0-2 S: 0-2
R.Studzinska
180515_AP_3 7 (0.194) Cm (6:9-15:30)

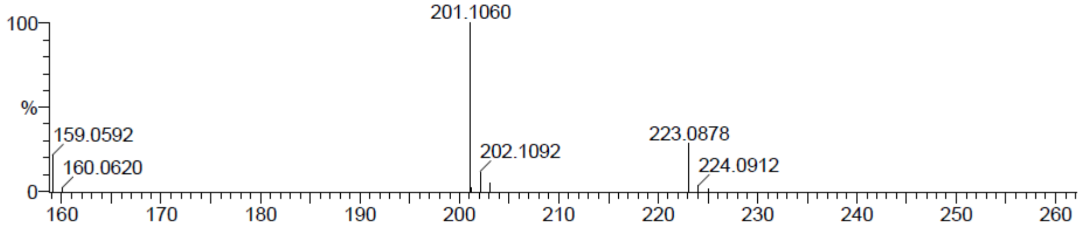

Minimum:				-1.5				
Maximum:		15.0	10.0	50.0				
Mass	Calc. Mass	mDa	PPM	DBE	i-FIT	Norm	Conf(%)	Formula
201.1060	201.1062	-0.2	-1.0	2.5	56.2	n/a	n/a	C9 H17 N2 O [illegible]

S3.3. HRMS spectra of compounds **3c**

Elemental Composition Report

3d

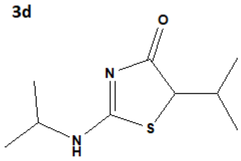

Page 1

Single Mass Analysis

Tolerance = 10.0 PPM / DBE: min = -1.5, max = 50.0
Element prediction: Off
Number of isotope peaks used for i-FIT = 3

Monoisotopic Mass, Even Electron Ions
65 formula(e) evaluated with 1 results within limits (up to 50 closest results for each mass)
Elements Used:
C: 0-15 H: 0-20 N: 0-3 O: 0-2 S: 0-2
R.Studzinska
180515_AP_4 7 (0.194) Cm (6:9-15:30)

1: TOF MS ES+
1.29e+007

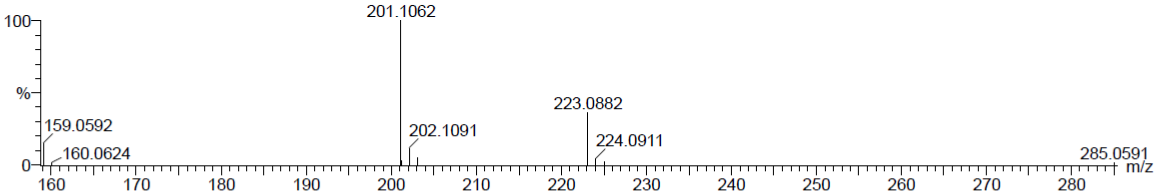

Minimum:				-1.5				
Maximum:		15.0	10.0	50.0				
Mass	Calc. Mass	mDa	PPM	DBE	i-FIT	Norm	Conf(%)	Formula
201.1062	201.1062	0.0	0.0	2.5	54.4	n/a	n/a	C9 H17 N2 O S

S3.4. HRMS spectra of compounds **3d**

Elemental Composition Report

Page 1

3e

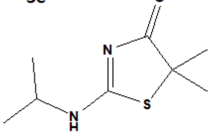

Single Mass Analysis

Tolerance = 10.0 PPM / DBE: min = -1.5, max = 50.0
Element prediction: Off
Number of isotope peaks used for i-FIT = 3

Monoisotopic Mass, Even Electron Ions
66 formula(e) evaluated with 1 results within limits (up to 50 closest results for each mass)
Elements Used:
C: 0-15 H: 0-20 N: 0-3 O: 0-2 S: 0-2
R.Studzinska
180515_AP_5 7 (0.194) Cm (7:8-(1:4+14:29))

1: TOF MS ES+
4.48e+006

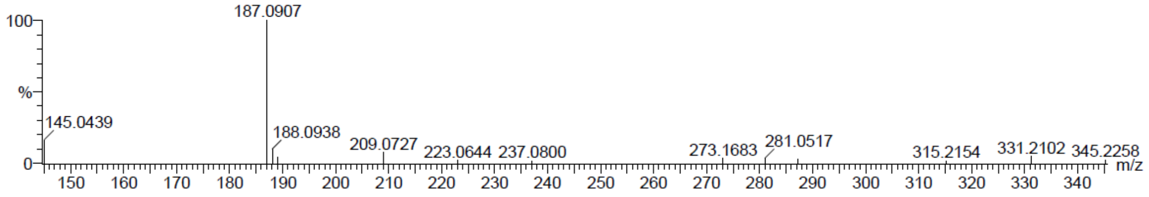

Minimum:				-1.5				
Maximum:		15.0	10.0	50.0				
Mass	Calc. Mass	mDa	PPM	DBE	i-FIT	Norm	Conf(%)	Formula
187.0907	187.0905	0.2	1.1	2.5	30.7	n/a	n/a	C8 H15 N2 O S

S3.5. HRMS spectra of compounds **3e**

Elemental Composition Report

Page 1

3f

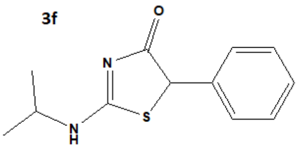

Single Mass Analysis

Tolerance = 10.0 PPM / DBE: min = -1.5, max = 50.0
Element prediction: Off
Number of isotope peaks used for i-FIT = 3

Monoisotopic Mass, Even Electron Ions
51 formula(e) evaluated with 1 results within limits (up to 50 closest results for each mass)
Elements Used:
C: 0-15 H: 0-20 N: 0-3 O: 0-2 S: 0-2
R.Studzinska
180515_AP_8 7 (0.194) Cm (6:9-(1:3+12:30))

1: TOF MS ES+
7.53e+005

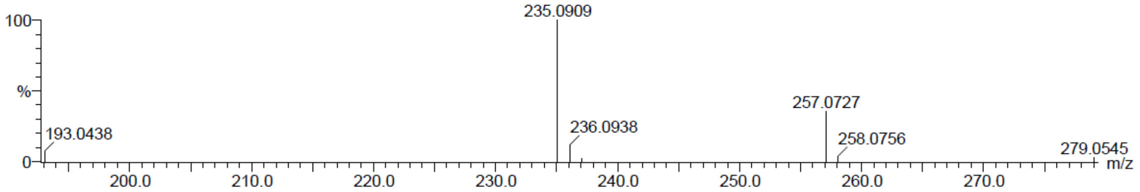

Minimum:				-1.5				
Maximum:		15.0	10.0	50.0				
Mass	Calc. Mass	mDa	PPM	DBE	i-FIT	Norm	Conf(%)	Formula
235.0909	235.0905	0.4	1.7	6.5	34.2	n/a	n/a	C12 H15 N2 O S

S3.6. HRMS spectra of compounds **3f**

Elemental Composition Report

3g

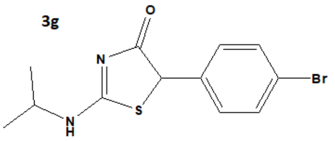

Page 1

Single Mass Analysis

Tolerance = 10.0 PPM / DBE: min = -1.5, max = 50.0
Element prediction: Off
Number of isotope peaks used for i-FIT = 3

Monoisotopic Mass, Even Electron Ions
171 formula(e) evaluated with 1 results within limits (up to 50 closest results for each mass)
Elements Used:
C: 0-15 H: 0-20 N: 0-3 O: 0-2 S: 0-2 Br: 0-8

R.Studzinska
180515_AP_9 7 (0.194) Cm (5:11-(1:4+13:30))

1: TOF MS ES+
3.80e+006

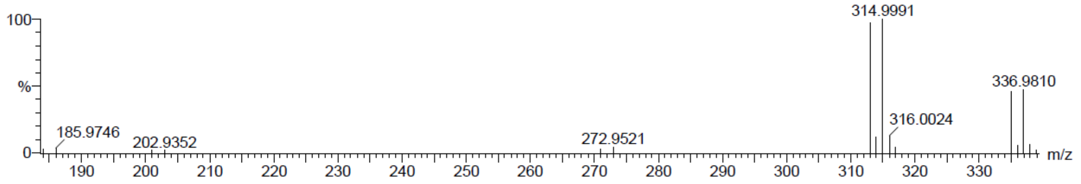

Minimum:				-1.5				
Maximum:		15.0	10.0	50.0				
Mass	Calc. Mass	mDa	PPM	DBE	i-FIT	Norm	Conf(%)	Formula
313.0011	313.0010	0.1	0.3	6.5	28.9	n/a	n/a	C12 H14 N2 O S Br

S3.7. HRMS spectra of compounds **3g**

Elemental Composition Report

3h

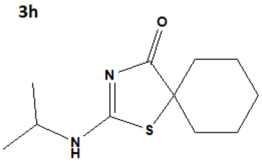

Page 1

Single Mass Analysis

Tolerance = 10.0 PPM / DBE: min = -1.5, max = 50.0
Element prediction: Off
Number of isotope peaks used for i-FIT = 3

Monoisotopic Mass, Even Electron Ions
58 formula(e) evaluated with 1 results within limits (up to 50 closest results for each mass)
Elements Used:
C: 0-15 H: 0-20 N: 0-3 O: 0-2 S: 0-2
R.Studzinska
180515_AP_6 7 (0.194) Cm (6:9-(15:30+1:4))

1: TOF MS ES+
2.86e+007

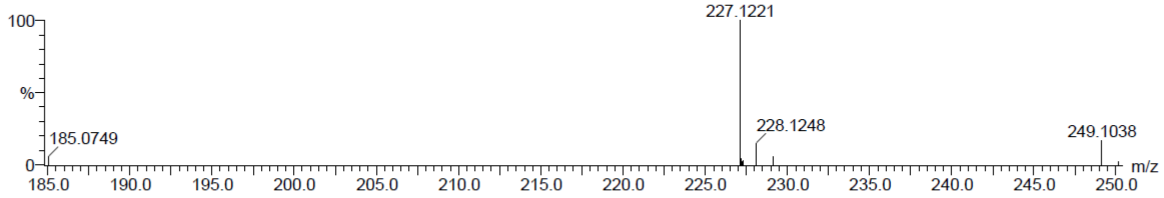

Minimum:				-1.5				
Maximum:		15.0	10.0	50.0				
Mass	Calc. Mass	mDa	PPM	DBE	i-FIT	Norm	Conf(%)	Formula
227.1221	227.1218	0.3	1.3	3.5	138.6	n/a	n/a	C11 H19 N2 O S

S3.8. HRMS spectra of compounds **3h**

Elemental Composition Report

3i

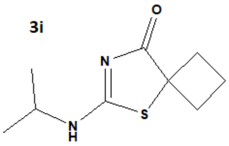

Single Mass Analysis

Tolerance = 10.0 PPM / DBE: min = -1.5, max = 50.0
Element prediction: Off
Number of isotope peaks used for i-FIT = 3

Monoisotopic Mass, Even Electron Ions
65 formula(e) evaluated with 1 results within limits (up to 50 closest results for each mass)
Elements Used:
C: 0-15 H: 0-20 N: 0-3 O: 0-2 S: 0-2

R.Studzinska
180515_AP_7 7 (0.194) Cm (6:9-(1:3+16:30))

1: TOF MS ES+
1.47e+007

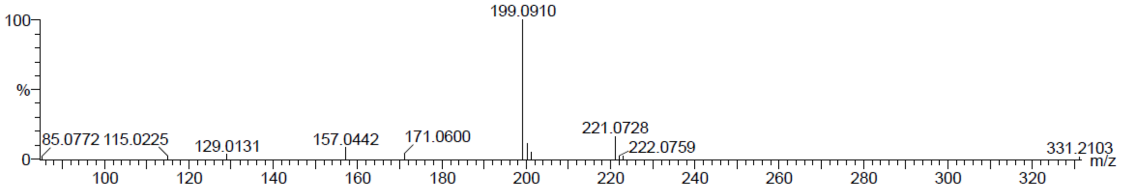

Minimum:				-1.5				
Maximum:		15.0	10.0	50.0				
Mass	Calc. Mass	mDa	PPM	DBE	i-FIT	Norm	Conf(%)	Formula
199.0910	199.0905	0.5	2.5	3.5	56.7	n/a	n/a	C9 H15 N2 O S

S3.9. HRMS spectra of compounds **3i**
